# The Combined Role of Silanols and Oxidative Stress in Determining Engineered Stone Dust Toxicity

**DOI:** 10.1021/acsorginorgau.5c00089

**Published:** 2025-11-05

**Authors:** Cristina Pavan, Marianna Fimiani, Stefania Cananà, Aleandro Diana, Matteo Marafante, Stefano Bertinetti, Guillermo Escolano-Casado, Lorenzo Mino, Dino Pisaniello, Riccardo Leinardi, Maura Tomatis, Francesco Turci

**Affiliations:** † Department of Chemistry, 9314 University of Turin, Turin 10125, Italy; ‡ “G. Scansetti” Interdepartmental Centre for Studies on Asbestos and Other Toxic Particulates, University of Turin, Turin 10125, Italy; § Louvain Centre for Toxicology and Applied Pharmacology, Institut de Recherche Expérimentale et Clinique (IREC), 83415 Université catholique de Louvain, Brussels 1200, Belgium; ∥ Adelaide Exposure Science and Health, School of Public Health, University of Adelaide, Adelaide 5005, Australia; ⊥ Department of Veterinary Sciences, University of Turin, Grugliasco 1095, Italy

**Keywords:** engineered stone, artificial stone, composite, silica, silanol, oxidative stress, membrane

## Abstract

Engineered stone (ES) silicosis is emerging as a global occupational health crisis, caused by exposure to respirable particles generated during the processing of ES composite materials. ES composites comprise crystalline silica (predominantly quartz), inorganic aggregates, polymeric resins, and pigments. The severity of lung disease in workers contrasts with the modest effects observed in short-term in vitro studies, exposing a critical gap in our mechanistic understanding of ES dust toxicity. In this work, we examined the surface chemistry and reactivity of ES dust obtained from a slab with high crystalline silica content, before and after incubation (up to two months) in simulated lung fluids: artificial lysosomal fluid (ALF, pH ∼ 4.5) and lung lining fluid simulant (Gamble’s solution, GS, pH ∼ 7.4). Damage to model membranes (red blood cell, RBC), an initiating event in ES-induced toxicity, was quantified by membranolytic assay. Pristine ES dust was negligibly membranolytic. Incubation in ALF markedly increased ES membranolytic activity, correlating with partial degradation of the resin. A complete removal of the resin produced a dust with further enhanced activity, associated with the exposure of nearly free silanol (NFS) groups, a recognized molecular trigger of quartz toxicity. NFS were detected by infrared spectroscopy after H/D isotopic exchange. ALF incubation also led to substantial release of transition metal ions, which catalyzed the formation of hydroxyl and carboxyl radicals, detected by EPR spectroscopy. In contrast, GS exposure resulted in minimal membranolytic activity and low radical generation. Our findings suggest that prolonged residence of ES dust in lung cellular environments, particularly lysosomes, promotes resin degradation, exposes reactive silanols, and releases transition metal ions, thereby imparting both membranolytic and oxidative potential. This work provides new molecular insight into ES dust toxicity, emphasizes the urgency of safer occupational practices, and paves the way to safe-by-design strategies for future composite materials.

## Graphical abstract

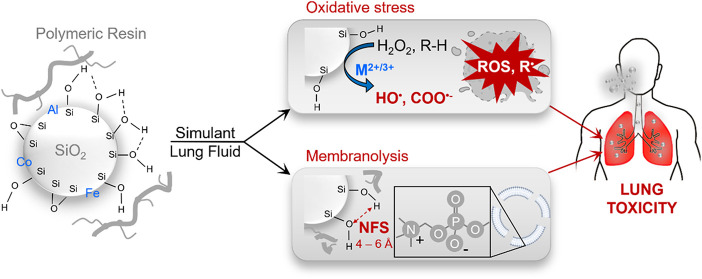

## 1 Introduction

Engineered stone (ES) has become very popular over the last 20 years for benchtops, flooring, wall facing, and other applications. However, its use has led to growing occupational lung disease among workers, including silicosis^1,2^ and development of autoimmune rheumatic disease,^3^ due to exposure of respirable dust generated during ES cutting, grinding and polishing. The onset of ES-related pathologies currently represents a global occupational health issue, with clusters of silicosis and lung abnormalities reported in Spain,^4,5^ Italy,^6,7^ Belgium,^8^ Israel,^9^ Australia,^10,11^ USA,^12,13^ UK,^14^ and China.^15,16^ Clinical evidence suggests that the disease associated with respirable ES dust is unusually aggressive. This form of accelerated silicosis is distinguished from traditional silicosis by a reduced dust exposure duration and a shorter latency period.^4,16,17^ The pathogenesis of ES-associated silicosis has been attributed not only to the high levels of exposure to dust containing crystalline silica released during ES processing and installation,^12,18–21^ but also to some physicochemical characteristics unique to ES dust.^19,21–24^ ES is an artificial composite made up of crystalline silica (SiO_2_) up to 90 wt %, inorganic aggregates and pigments, and an organic polymeric resin.^22^ Respirable crystalline silica (RCS), including quartz and cristobalite particles, is a well-known carcinogenic agent.^25,26^ The presence of additional elements, including Al, Fe, Cu, Co and Ti, has been reported and possibly related to the high capacity of these materials to induce oxidative stress.^19,21–24,27^ Moreover, volatile organic compounds (VOCs) and polycyclic aromatic hydrocarbons (PAHs), which are released during cutting operations, have been suggested to contribute to the inflammatory reaction and respiratory pathology associated with ES dust.^20,21^ Besides the chemical composition of the dust released during the cutting of ES, morphology, size distribution, and surface chemistry of the particles can affect toxic effects of ES dust.^28–30^

However, the aggressiveness of the disease induced by ES dust contrasts with pioneer *in vitro* studies from our group on murine alveolar macrophages (MH-S), human bronchial epithelial cells (BEAS-2B), and model membranes (red blood cells), which revealed a negligible or very low cytotoxic effects for ES dusts collected in the workplace.^22^ Further *in vitro* studies on a larger set of ES dusts confirmed the low cytotoxic effects on human macrophages (THP-1) and alveolar epithelial cells (A549), and only a few among the investigated ES dust samples induced inflammatory cytokine release.^23,31^ Notably, albeit the emergence of ES-associated silicosis has been recognized for over a decade, very few *in vitro* studies – and no *in vivo* studies – have tried to elucidate the mechanisms underlying ES toxicity and specific molecular pathways.

We hypothesized that the peculiar composition and surface chemistry of ES dust might play a role in the discrepancy between the severity of the observed disease and the relatively low *in vitro* toxicity. In particular, we challenged the hypothesis that the presence of the polymeric resin that binds quartz particle in the final products may mask the specific sites of crystalline silica surface, which are known to mediate interactions with cell membranes.^22,32^ An analogous inhibitory effect was described for organic treatments aimed at reducing RCS short-term toxic responses, such as organosilane coatings^33–35^ or adsorption of polymers (e.g., poly­(2-vinylpyridine-N-oxide)).^36,37^ The inhibitory mechanism proposed for RCS coated with polymers is that silanols groups (≡Si–OH) present on silica surfaces are masked by the organic residues. Notably, the presence of nearly free silanols (NFS) at silica surface has been associated with the capacity of quartz or cristobalite particles to damage biomembranes^38^ and induce short and long-term pathologic response in the lungs of rodents.^39^ Because of their specific spatial configuration, NFS are energetically favored in interacting with zwitterionic phospholipid headgroups, promoting membrane damage.^40,41^ The disruption of the cell membrane is considered a critical event in the onset of lung inflammation and the development of silicosis.^42^ Upon internalization into alveolar macrophages, RCS particles may destabilize the phagolysosome membrane, triggering a cascade of proinflammatory and profibrotic responses through the activation of the inflammasome and the caspase-1-dependent pathway.^43^

Compared to pure quartz dust, ES dust is characterized by ca. 10 wt % polymeric resin and a non-negligible content of redox-active transition metals, which may contribute to the generation of reactive oxygen species (ROS), that in turn promote cellular oxidative stress, and the activation of inflammatory pathways, eventually exacerbating lung cell damage.^22–24,44^

This study aims to elucidate the mechanistic role of ES particle surface properties in (i) eliciting cell membrane damage, and (ii) inducing oxidative stress, two key molecular initiating events (MIE) in inhaled particle toxicity. From consolidated studies on RCS, these two key events are related to NFS and generation of free radical species catalyzed by redox active species, respectively.^40,45^ To take in consideration the simultaneous presence of crystalline silica, resin, and metal oxides in ES dust, we hypothesized that ES pro-inflammatory properties may undergo alterations upon exposure to pulmonary or cellular fluids, potentially giving rise to modified or additional molecular mechanisms of toxicity. Thus, membranolytic activity, resin content, surface chemical groups, and capacity to generate hydroxyl (^•^OH) and carboxyl (^•^COO^–^) radicals were assessed on a set of ES dust samples incubated in simulated lung fluids (SLF). Two SLF were used: artificial lysosomal fluid (ALF), representative of the acidic environment within the lysosome organelles where particles accumulate upon macrophage phagocytosis, and Gamble’s solution (GS), representative of the lung lining fluid at a physiological pH.^46,47^ A thermal oxidation treatment (i.e., calcination) was performed to accelerate resin degradation. The membranolytic activity was tested against red blood cells (RBCs). RBCs are not involved in the pathogenesis of RCS or particles in general, yet they serve as a straightforward model for investigating particle–membrane interactions and assessing particle surface reactivity.^48^

## 2 Experimental Section

### 2.1 Engineering Stone (ES) and Quartz Dusts

The main ES dust sample analyzed in this study was derived from a slab with a high crystalline silica content (>90 wt %), hereafter referred to as **ES1**. The dust was generated by dry cutting, using a diamond saw blade. This dry cutting procedure was selected to reproduce standard conditions in real occupational scenarios, where ES dust is mainly generated by dry mechanization processes. The ES1 slab was fabricated using a standard formulation, except that dyes and pigments were omitted to minimize potential contamination and material chemical variability due to the industrial use of several organic and inorganic dyes and pigments, thereby isolating the effects of the main ES components. Specifically, the slab was prepared with a mixture containing only quartz (particle size distribution ranging from 1 to 400 μm), an unsaturated polyester resin, and a cobalt-based polymerization catalyst. A limitation of using this *ad hoc* prepared slab is that it does not allow the assessment of effects potentially related to the organic and inorganic pigments commonly used in commercial ES fabrication. The quartz dust (**Qz**) was obtained by sieving (30 μm mesh sieve on a vibrating apparatus) the finest fraction of the raw quartz (grain size: 1–24 μm) used to produce ES1 composite. The polymeric resin dust (**resin**) was obtained by dry cutting a polyester slab made of the same resin used in the production of ES1 composite.

An additional set of engineered stone dust samples (**ES2–9**) with high crystalline silica content was used only to test the ALF-induced resin degradation and the consequent variation in hemolytic activity. These dust samples were obtained from commercially available products and had been previously prepared and characterized in an earlier study.^44^ Briefly, the slabs were first cut using a wet diamond blade saw, then crushed using a tungsten carbide (WC) jaw crusher and a ring mill for 4 min. Although this milling process does not reproduce the cutting operations in occupational settings, it yielded dusts containing respirable and inhalable-size particles.^44^

The commercial quartz Min-U-Sil 5 (US Silica Company, Berkely Springs, WV) was used as positive reference particle (**rQz**) in membranolysis tests because of its well-documented membranolytic and toxicity effects.^39^

### 2.2 Chemical Reagents

When not otherwise specified, all reagents were of analytical grade and purchased from Merck (Sigma-Aldrich). The water used was ultrapure milli-Q water (Merck-Millipore). RBCs were obtained from sheep blood in Alsever’s solution (Oxoid). 5,5′-Dimethyl-1-pirroline-N-oxide (DMPO) was purchased from Cayman Chemical Company (Ann Arbor).

### 2.3 Incubation in SLF

The chemical composition of ALF and GS refers to Colombo et al.^46^ and is described in Table 1. The main differences between GS and ALF are the acidity (pH 7.4 and pH 4.5, respectively) and the organic content (ALF has much higher organic content than GS). To avoid microbial growth and mold formation, 0.02% formaldehyde was added to both the solutions. The pH was adjusted with some drops of 1 M NaOH or HCl (ALF, pH 4.5 ± 0.1; GS, pH 7.4 ± 0.1).

**Table 1:** Composition (g/l) of ALF and GS

**chemicals**	**ALF** (g/L)	**GS** (g/L)
magnesium chloride, MgCl_2_	0.050	0.095
sodium chloride NaCl	3.21	6.019
potassium chloride, KCl		0.298
disodium hydrogen phosphate, Na_2_HPO_4_	0.071	0.126
sodium sulfate, Na_2_SO_4_	0.039	0.063
calcium chloride dihydrate, CaCl_2_·2H_2_O	0.128	0.368
sodium acetate, C_2_H_3_O_2_Na		0.574
sodium hydrogen carbonate, NaHCO_3_		2.604
sodium citrate dihydrate, C_6_H_5_Na_3_O_7_·2H_2_O	0.077	0.097
citric acid, C_6_H_8_O_7_	20.8	
sodium hydroxide, NaOH	6.00	
sodium tartrate dihydrate, C_4_H_4_O_6_Na_2_·2H_2_O	0.090	
sodium pyruvate, C_3_H_3_O_3_Na	0.086	
sodium lactate, C_3_H_5_NaO_3_	0.085	
glycine, H_2_NCH_2_COOH	0.059	

The protocol for incubation is described in Maharjan et al.,^44^ with minor modifications. Five grams of ES dust was mixed with 250 mL of ALF or GS in a Schott bottle. The bottles containing a mixture of ES dust and SLF were placed in a horizontal shaking stirrer (ASAL s.r.l.) at 37 °C and gently agitated at 120 rpm. Aliquots (30 mL) of fluids were extracted periodically at 1 week, and two, four and 8 weeks of incubation and were filled again with 30 mL new ALF. To avoid transfer of particles, the extracted aliquot was centrifuged (10,000*g*, 25 °C, 3 min, Rotina 380R, Hettich Instruments) and the resulting pellet was returned to the original bottles. At the end of the incubation time, the suspensions were transferred to falcon tubes and centrifuged. Then the powder was washed 3 times with milli-Q water and dried overnight in an oven (60 °C). Each experiment was conducted in duplicates and blank experiments (containing only SLF) were performed.

### 2.4 Membranolytic Activity

The protocol for assessing the membranolytic activity, i.e., hemolytic activity, of particles refers to previous studies.^49,50^ Briefly, RBCs were purified from sheep blood in Alsever’s solution by centrifugation (3000*g*, 2 min, Rotina 380R, Hettich Instruments) and washing three times with 0.9% NaCl water solution. RBCs were suspended in 0.01 M phosphate buffered saline (PBS) at the concentration of 5% by volume. Dust samples were dispersed at the initial concentration of 30 mg/mL in 0.01 M PBS and sonicated in an ultrasound bath (Falc, Italy) for 2 min, just before testing. Serial dilutions of the starting dispersion were performed according to the final doses used for experiments. Negative and positive controls consisted of 0.01 M PBS and 0.1% Triton-X 100 in PBS, respectively. Particle dispersions were incubated with RBCs on a horizontal shaking stirrer (ASAL s.r.l.) for 30 min and centrifuged at 216*g* for 5 min. Supernatants were transferred to a new plate, and the absorbance of the hemoglobin released was determined at 540 nm on a UV/vis spectrophotometer (Ensight, Perkin-Elemer) using the software Kaleido 3.0 (Perkin-Elemer).

### 2.5 Determination of Resin Content

Thermogravimetric analysis (TGA) was applied to assess resin content in ES dusts. Approximately 20 mg of dust was heated in artificial air flow (O_2_ 35 mL/min and N_2_ 65 mL/min) up to 900 °C at the rate of 10 °C/min. Weight loss after sample combustion was determined by an ultramicrobalance (sensitivity: 0.1 μg) (Pyris 1 TGA, PerkinElmer).

### 2.6 Assessment of Surface Chemical Groups and Silanol Distribution

Diffuse Reflectance Infrared (DRIFT) spectroscopy was used to determine the surface chemical groups and silanol distribution of the particles, following a method previously described.^40,51^ Briefly, a Spectra-Tech diffuse reflectance unit, equipped with an environmental chamber connected to a conventional vacuum line (residual pressure, ≤ 1 × 10^–4^ mbar), was used to carry out all desorption/adsorption experiments *in situ*. The spectra were collected on the powders (ca. 50 mg) with a Bruker Vector22 FTIR spectrometer (Globar source, MCT detector; resolution, 4 cm^–1^) averaging 128 scans for spectrum to obtain a good signal-to-noise ratio. The spectra were recorded as such and after H/D isotopic exchange by adsorption/desorption of D_2_O vapors (Sigma-Aldrich; 99.90% D) in order to convert surface OH in the OD form.

### 2.7 Thermal Oxidation Treatment of Dust

To obtain ES1–400 °C and Qz-400 °C, the dust samples were heated in a muffle furnace with a ramp rate of 10 °C/min and held isothermally at 400 °C for 2 h, then allowed to cool to room temperature (r.t.) inside the furnace. The selected temperature and protocol were based on the TGA of ES1, indicating complete resin degradation at this value.

### 2.8 Elemental Analysis of Leachates and Speciation Model

Elemental analysis was performed using Inductively Coupled Plasma-Optical Emission Spectroscopy (Agilent 5110 ICP-OES), to quantify the metal release in the leachates of dust incubated in SLF after 1 h, 1 month and 2 months. The protocol of dust incubation in SLF was slightly modified to avoid metal loss. The dust (600 mg) was incubated in ALF and GS (15 mL) without media refreshing, under gentle agitation at 120 rpm using a horizontal shaking incubator at 37 °C. At the end of each incubation time, the supernatant (10 mL) was removed and stored at 4 °C for ICP-OES analysis. Al, Ca, Co, Fe, Mg and Si were analyzed, based on the elemental composition of ES1, quartz, and resin dust (Supporting Information, Table S1). The calibration curve was carried out following the external calibration method. Metal standard solutions were prepared from concentrated (1000 mg/L) stock solutions (CPI International) in milli-Q water and acidified with 0.1% nitric acid (65% HNO_3_), to guarantee the titles of the solution preventing metal precipitation. Dust supernatant and the blank solutions (i.e., ALF and GS alone) were diluted 1:10 and acidified with 0.1% HNO_3_. One of the standard solutions was analyzed at regular intervals over time during the instrumental analysis to check and correct the instrumental drift. The contribution from the blank reference sample (matrix effects) was subtracted from the concentrations of the elements in dust supernatants. Three instrumental replicates and two independent experiments were performed. The limit of detection (LOD) was 0.2 mg/L for Ca, 0.1 mg/L for Al and Mg, 0.05 mg/L for Fe, 0.04 mg/L for Si and 0.03 mg/L for Co. The distribution of species in the leachates obtained from ALF and GS was calculated by the open-source software PyEs for the calculation of the concentration of the species in solution at the thermodynamic equilibrium (Supporting Information, Supporting methods).^52^

### 2.9 Detection of Free Radicals

Electron Paramagnetic Resonance (EPR) spectroscopy coupled with the spin trapping technique was employed to assess the formation of hydroxyl (^•^OH) and carboxyl (^•^COO^–^) radicals in ALF or GS. The protocol is described in previous studies, with modifications related to the media used and incubation time.^22,53^ To assess ^•^OH formation, the dust (40 mg/mL) was suspended in ALF or GS, DMPO (34 mM) as spin trapping agent, and H_2_O_2_ (80 mM) as target molecule. To assess ^•^COO^–^ formation, the dust (40 mg/mL) was suspended in ALF or GS, DMPO (34 mM) and the target molecule sodium formate (1 M). The mixtures were incubated at 37 °C with gentle agitation (120 rpm) on a horizontal shaking stirrer (ASAL s.r.l.) for 10, 30, and 60 min. The mixtures were then centrifuged at 10,000*g* for 30 s (Rotina 380R, Hettich). An aliquot (50 μL) of the supernatant was then collected using a glass microcapillary, and the EPR spectra were recorded using a MiniScope 100 spectrometer (Magnettech). The formation of ^•^OH and ^•^COO^–^ from ES1 dust leachate (ES1-L) were evaluated by incubating the dust (40 mg/mL) in ALF or GS for 60 min. After the incubation, the samples were centrifuged at 10,000*g* for 30 s to separate the particulate fraction from the leachate. Then the leachate was added to DMPO (34 mM) and H_2_O_2_ (80 mM) or sodium formate (1 M). Each experimental condition was repeated in at least two independent experiments. The EPR spectra were double integrated to obtain quantitative data with OriginPro 2023 Academic (OriginLab Corporation) software.

### 2.10 Statistics

Statistical parameters, including the number of independent experiments and statistical significance, are reported in the figures and figure legends. Data were analyzed by one-way ANOVA followed by Šídák’s or Dunnett’s post hoc test, as appropriate. In all tests, a 95% confidence interval was used, for which differences with *p* < 0.05 were considered statistically significant. Data are presented as mean ± standard error of the mean (SEM) or standard deviation (SD). Statistical analysis was performed with the GraphPad Prism 10 software (Siemens Digital Industries Software).

## 3 Results and Discussion

### 3.1 Incubation of ES Dust in a Simulated Lung Fluid Induced Resin Degradation and Increased Membranolysis

To assess surface reactivity, the membranolytic activity of the engineered stone dust (ES1) was assessed as its capacity to induce hemolysis (%) in sheep RBCs (Figure 1a). The mineralogical and elemental composition of ES1 is reported the Supporting Information, Table S1. ES1 dust is characterized by a high content of crystalline silica (ca. 85 wt % quartz, Table S1), albite (9 wt %), and some metal impurities, including Fe, Al, Ti, and Zr (Table S1) due to natural trace mineral impurities or contamination from the cutting tool. ES1 morphology was typical of particles obtained by fracturing, showing conchoidal fractures (Figure S1), and particle size in the respirable range (<4 μm)^54^ (Figure S2). A relatively pure commercial quartz was used as a positive reference particle (rQz) because of its well-established membranolytic and toxic effects.^39^ ES1 dust exhibited no hemolytic activity at any of the investigated doses, with all values below 2%, a threshold considered indicative of hemocompatibility.^55^ In contrast, the positive reference quartz (rQz) displayed a strong, dose-dependent increase in hemolytic activity at the same doses of ES1. This finding confirms the results obtained in previous studies for as-processed ES dust of variegate composition, which similarly showed a negligible hemolytic activity.^22,56^ The membranolytic activity of ES1 was further tested after a 2-months incubation in ALF, a simulant fluid that mimics the acidic environment (pH 4.5) within the lysosomes.^46,47^ Notably, after ALF incubation, the hemolytic activity of ES1 increased, showing a significant difference with respect to pristine ES1 at the highest doses of particles (2.5, 5, and 10 mg/mL) (Figure 1a). This increase was not attributed to a variation in the specific surface area (SSA) of ES1, a key parameter that could influence particle hemolysis, as the SSA on the contrary slightly reduced after incubation in ALF (Table S2).

**Figure 1:**
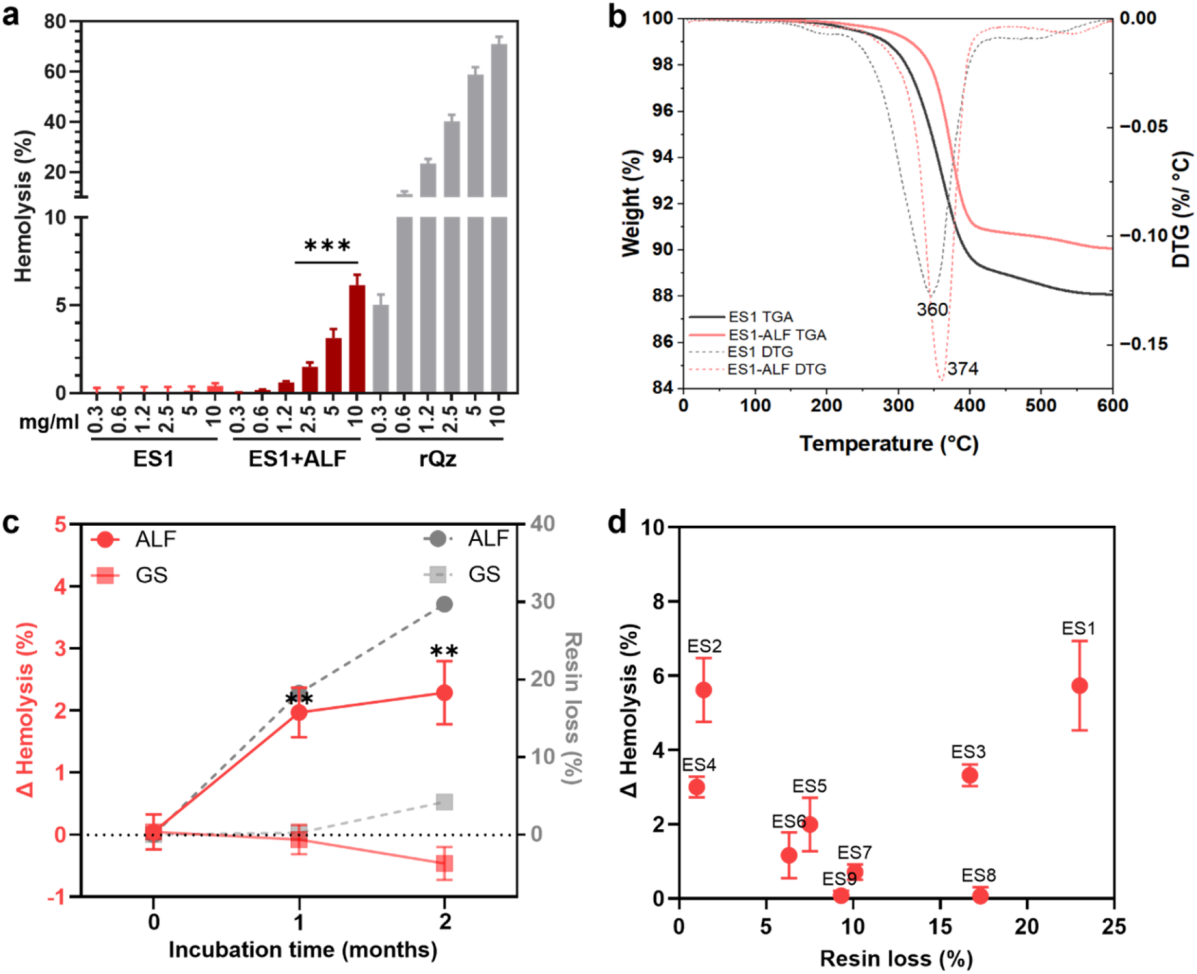
Membranolytic activity and resin loss of ES dust incubated in simulated body fluids. (a) Membranolytic activity (hemolysis, %) at increasing particle concentration (mg/mL) of pristine ES1 dust, ES1 dust after incubation for 2 months in ALF, and a reference quartz (rQz). Data are mean ± SEM of three independent experiments. One-way ANOVA and Šídák’s *post hoc* test were applied to compare ES1 vs ES1+ALF for each particle dose, ***p < 0.001. (b) Thermogram (solid lines) and derivative curves (dotted lines) obtained by TGA analysis in artificial air of pristine ES1 dust and ES1 dust after incubation for 2 months in ALF. (c) Solid red lines: difference of membranolytic activity (ΔHemolysis, %) before and after incubating ES1 (10 mg/mL) in ALF and GS for 1 and 2 months. Data are mean ± SEM of three independent experiments. One-way ANOVA with Dunnet’s *post hoc* test was applied to compare ΔHemolysis (%) at 1 and 2 m vs 0 m, **p < 0.01. Dotted gray lines: resin loss (%) assessed by TGA analysis before and after incubating ES1 (10 mg/mL) in ALF and GS for 1 and 2 months. (d) Difference of membranolytic activity (ΔHemolysis, %) before and after particle incubation in ALF for 2 months (at 10 mg/mL) plotted against the resin loss (%) assessed by TGA analysis before and after particle incubation in ALF for 2 months for a set of eight ES samples (ES1–8) of different origin and composition.

In previous studies, we assigned the negligible membranolytic activity of crystalline silica-rich ES dust to the presence of the organic polymeric resin, possibly masking crystalline silica surface reactive sites.^22,56^ Similarly, a reduction of the hemolytic and macrophage stimulatory activity has been reported when quartz was coated with polymers and organosilanes.^33–37^ We therefore hypothesized that the observed increase in hemolysis after ALF incubation was due to the degradation and partial loss of the resin, which could in turn expose membranolytic sites on the quartz surface. We measured by TGA analysis the resin content and thermal behavior of ES1 before and after 2 months incubation in ALF (Figure 1b). The dust was heated up to 600 °C in artificial air atmosphere (3:1 N_2_ and O_2_ mixture). The thermogram of ES1 showed an overall weight loss of ca. 12 wt % and was characterized by one main process. The derivative curve (DTG) of ES1 showed a well-defined minimum peak at 360 °C for a process starting at about 200 °C and ending at ca. 440 °C, where a second minor process took place. The total weight loss of pristine ES1 was in agreement with the nominal content of resin in the slab and with previous studies demonstrating that this process corresponds to the oxidative degradation of the polymeric resin.^19,22,57^ After incubation in ALF, ES1 exhibited a total weight loss of approximately 9 wt %, which was lower than that of the pristine material, suggesting partial resin removal from the particles during incubation. The DTG minimum peak shifted to a higher temperature (374 °C), possibly indicating alterations in the organic resin and its link to particle surface, requiring a higher temperature for matrix decomposition.

In addition to ALF, we incubated ES1 in GS, a simulant fluid representative of the interstitial fluid of the deep lung with a physiological pH (7.4).^46,47^ Figure 1c compares the effect of GS on the membranolytic activity (hemolysis) and resin loss (TGA analysis) of ES1 after 1 and 2 months of incubation with those observed after incubation in ALF. While incubation in ALF induced a time-dependent increase in both resin loss and membranolytic activity of ES1, GS incubation did not induce a significant variation in both hemolysis and resin loss, compared to the pristine sample. This suggests that the resin may be more labile under the peculiar ALF composition.

To check whether ALF incubation provoked an increased hemolysis in several ES dusts of different composition, an additional set of eight ES samples was incubated and then tested for hemolytic activity. The tested ES samples were from different origin and showed different minero-chemical composition (Table S1),^57^ but they were all characterized by a high content of crystalline silica (from 66 to 99 wt %). The set was tested for its hemolytic activity before and after 2-months incubation in ALF. The difference in membranolysis before and after incubation (ΔHemolysis% = Hemolysis% of dust post SBF – Hemolysis% of pristine dust, for the 10 mg/mL particle dose) is reported in Figure 1d and plotted against the resin loss %. After ALF incubation, the hemolytic activity increased by ca. 1–6% for most of the ES dusts investigated. Only two samples, ES8 and ES9, did not show any significant increase. The percentage of resin loss in ALF showed values ranging between 1 and 20% of the total resin. This finding indicates that even partial removal of resin resulted in an increased hemolytic activity of ES dust, possibly signaling that a few specific sites, including the membranolytic NFS, are involved in the activity of this class of compounds. The heterogeneous physicochemical properties of these dusts derived from commercially available ES slabs might account for the unpredictable effect of resin removal on the increase of hemolytic activity.

Overall, the present results pointed out that incubation of ES1 dust in ALF partially removed the resin, thereby increasing ES1 membranolysis. This effect occurred only in ALF and was also observed for some other ES dusts.

### 3.2 Resin Degradation by Thermal-Oxidation Treatment Induced a Strong Membranolytic Activity by ES Dust and Revealed NFS

To accelerate resin degradation and reproduce the cumulative effects of the prolonged oxidative and leaching processes that might occur in the dynamic environment of the lung, we thermally oxidized ES1 as a nonphysiological method to obtain resin-free dust particles. Considering that most of the resin is degraded at temperature below 400 °C (TGA analysis, Figure 1B) and that at higher temperatures the condensation of silanols occurs,^40^ we heated ES1 at 400 °C for 2h. Figure 2a shows the results of the thermal oxidation as a function of time and temperature. The prominent weight loss occurred within the first hour, when the temperature of 400 °C was achieved. During the 2h isotherm at 400 °C a slight weight loss still occurred. No further weight loss is observed when the temperature was raised above 400 °C, confirming that all the organic fraction is eliminated at 400 °C. This treatment induced 100% resin degradation (12 wt % of the total sample) and only slightly modified the SSA of ES1, which retained a low SSA typical of micron-sized dusts (Table S2). The same thermal oxidation treatment was applied on the pure quartz dust (Qz) that was used to produce ES1 slab. Qz exhibited a heterogeneous morphology similar to ES1, characterized by acute spikes and edges, and conchoidal fractures (Figure S1). The particle size distribution of Qz, with more than 95% of particles within the respirable range (Figure S2), and the SSA (1.7 m^2^/g, Table S2) were comparable to that of ES1. Thermal treatment on Qz clearly resulted in no weight loss (Figure S3).

**Figure 2:**
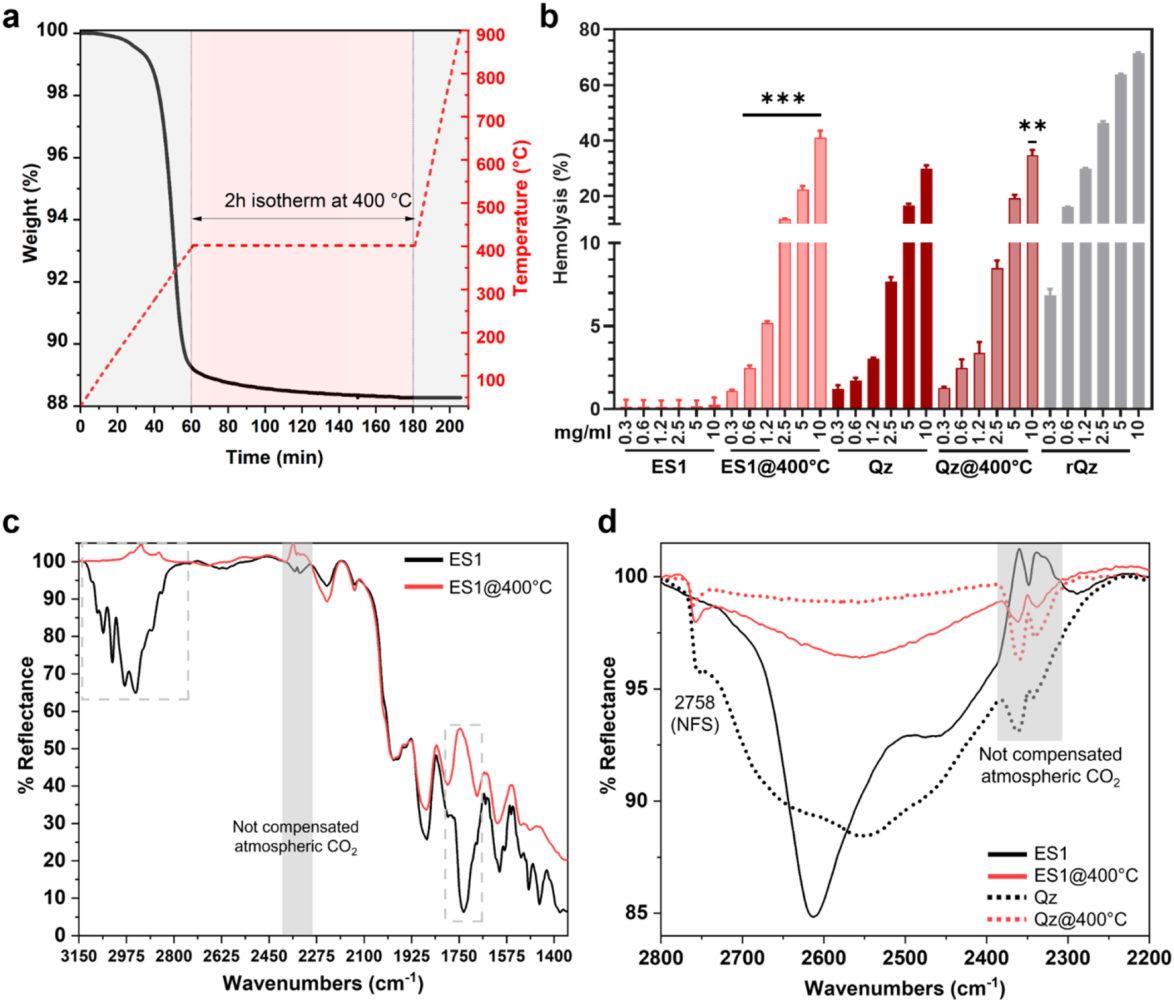
Membranolytic activity and surface chemistry of thermally treated ES dust. (a) Weight loss (%) of ES1 as a function of time and temperature as assessed by TGA analysis in artificial air. (b) Membranolytic activity (hemolysis, %) at increasing particle concentration (mg/mL) of pristine ES1 dust, ES1 dust heated at 400 °C for 2h (ES1@400 °C), the pure quartz (Qz), Qz heated at 400 °C for 2 h (Qz@400 °C), and a reference quartz (rQz). Data are mean ± SEM of three independent experiments. One-way ANOVA and Šídák’s *post hoc* test were applied to compare ES1 vs ES1@400 °C or Qz vs Qz@400 °C for each particle dose, **p < 0.01 and ***p < 0.001. (c) DRIFT spectra of pristine ES1 and ES1@400 °C. (d) DRIFT spectra in the 2800–2200 cm^–1^ range after H/D isotopic exchange of pristine ES1, ES1@400 °C, Qz and Qz@400 °C.

After resin degradation, the membranolytic activity of ES1 (ES1@400 °C) increased markedly across all investigated doses, reaching levels comparable to pristine quart (Qz) and quartz heated at 400 °C (Qz@400 °C) (Figure 2b). This suggests that resin removal exposes the pristine quartz surface, restoring the ability of silanols to interact with and damage RBC membranes. The increase in membranolytic activity after thermal oxidation was confirmed with two samples from the additional set of ES dusts (ES2 and ES4, Figure S4), indicating that heating is effective toward several types of high-crystalline silica ES dust. As expected, heating pure quartz at 400 °C (Qz@400 °C) did not significantly modify its membranolytic activity (Figure 2b), showing only a modest increase at the highest dose with respect to the pristine quartz (Qz), consistently with previous studies on quartz heated at similar temperatures.^40^

To determine whether the membranolytic activity of ES1 after thermal treatment is due to the presence of surface NFS, we comparatively investigated ES1 dust with IR spectroscopy in the diffuse reflectance (DRIFT) mode (Figure 2c). Pristine ES1 showed the presence of C–H stretching vibrations (νC–H) in the 3150–2750 cm^–1^ range, associated with both saturated and unsaturated groups, and of carbonyl ester group, which stretching vibration (νC=O) occurred at 1725 cm^–1^. Both these features are relative to the polymeric resin, which shows also two other bands at 1495 and 1455 cm^–1^. The other signals in the 2000–1500 cm^–1^ range could be assigned to overtones of bulk Si–O modes. Notably, the spectral features associated to νC–H and νC=O vibrations, which were assigned to the polymeric resin, completely disappeared after the thermal treatment (ES1@400 °C), while the signals of bulk Si–O modes remained. This finding confirms the efficacy of the selected thermal oxidation in completely removing the polymeric resin from ES dust.

To better resolve the presence of NFS, hydrogen–deuterium (H/D) isotopic exchange was performed (see Section 2), and the spectra were analyzed in the range associated with O–D stretching vibrations (νOD, 2800–2200 cm^–1^). The O–D spectra of ES1 (Figure 2d) showed two main bands at 2613 and 2465 cm^–1^. These signals are typical of the polymeric resin, indicating that the silanols at quartz surface (see spectrum of Qz sample in Figure 2d) either experience a strong interaction with the resin or are chemically modified by the resin itself. These bands completely disappear after the thermal treatment. Indeed, the spectrum of the heated ES1 (ES1@400 °C) showed a broad band between 2700–2400 cm^–1^, which is due to silanols mutually engaged in strong H-bonds, and a narrow peak at 2756 cm^–1^ assigned to NFS.^38,40,51^ This νOD pattern resembles the spectrum that has been typically found for pure quartz and silica in general.^38,40^ In fact, the surface silanol distribution of ES1@400 °C is comparable to the one observed for the membranolytic pure quartz (Qz) that made up ES1 treated at the same temperature (sample Qz@400 °C in Figure 2d). The lower intensity of the broad band due to H-bonded silanols in the Qz@400 °C with respect to ES1@400 °C suggested that silanol condensation, which may occur during thermal treatment at the selected temperature, was partially hindered in ES1@400 °C. This is possibly due to the presence of the resin which partly insulated the particle surface during the thermal degradation.

These experiments demonstrated that degradation of the resin through thermal oxidation exposed surface silanols, thereby increasing the availability of membranolytic NFS at the particle surface. Thus, silanol groups (and NFS) on ES dusts may become exposed following thermal, chemical or biochemical processes that remove the resin coating from the surface of quartz particles. The high membranolytic activity observed for both heated ES1 and pure quartz is likely due to the molecular interaction of NFS with the zwitterionic phosphocholine groups of membrane phospholipids, a mechanism that has been previously demonstrated for other quartz and silica particles.^40,41,58^

### 3.3 Incubation of ES dust in ALF Led to Metal Release and Free Radical Reactivity

Besides surface silanol groups, free radicals may generate oxidative stress in biological systems through mechanisms that involve transition metal ions that can catalyze Fenton and Fenton-like reactions.^45,53,59^ Thus, we used ICP-OES to quantify the metal content in the leachates from ES1, Qz, and the resin particles incubated for 1h, 1 and 2 months both in ALF (Figure 3a and Table S4) and in GS (Figure 3b and Table S5). Notably, in ALF (Figure 3a and Table S4) a higher concentration of metals was released with respect to GS. Fe and Co – potentially able to catalyze Fenton and Fenton-like reactions – were released in ALF in a time-dependent manner. At the same time points, higher concentrations of transition metal ions were leached from ES1, rather than from Qz. While Fe was detected by EDS in both ES1 and Qz pristine dust (Table S1), Co, being present only in trace amounts, was detected by ICP, which offers higher sensitivity. The presence of Co may originate from its use as a catalyst in resin polymerization or from the alloy of the cutting tool. Fe was also released from the resin dust, where it was likely present due to contamination from the cutting tool. Al occurred in the leachates of ES1 and Qz, because of the presence of albite (NaAlSi_3_O_8_) in both samples (Table S1). The leaching of Al, and Si to a minor extent, is relevant from a toxicological point of view as previous studies indicate the accumulation of those elements in the core of silicotic nodules from workers exposed to ES, suggesting a potential role of Al in nodule formation.^4^

**Figure 3:**
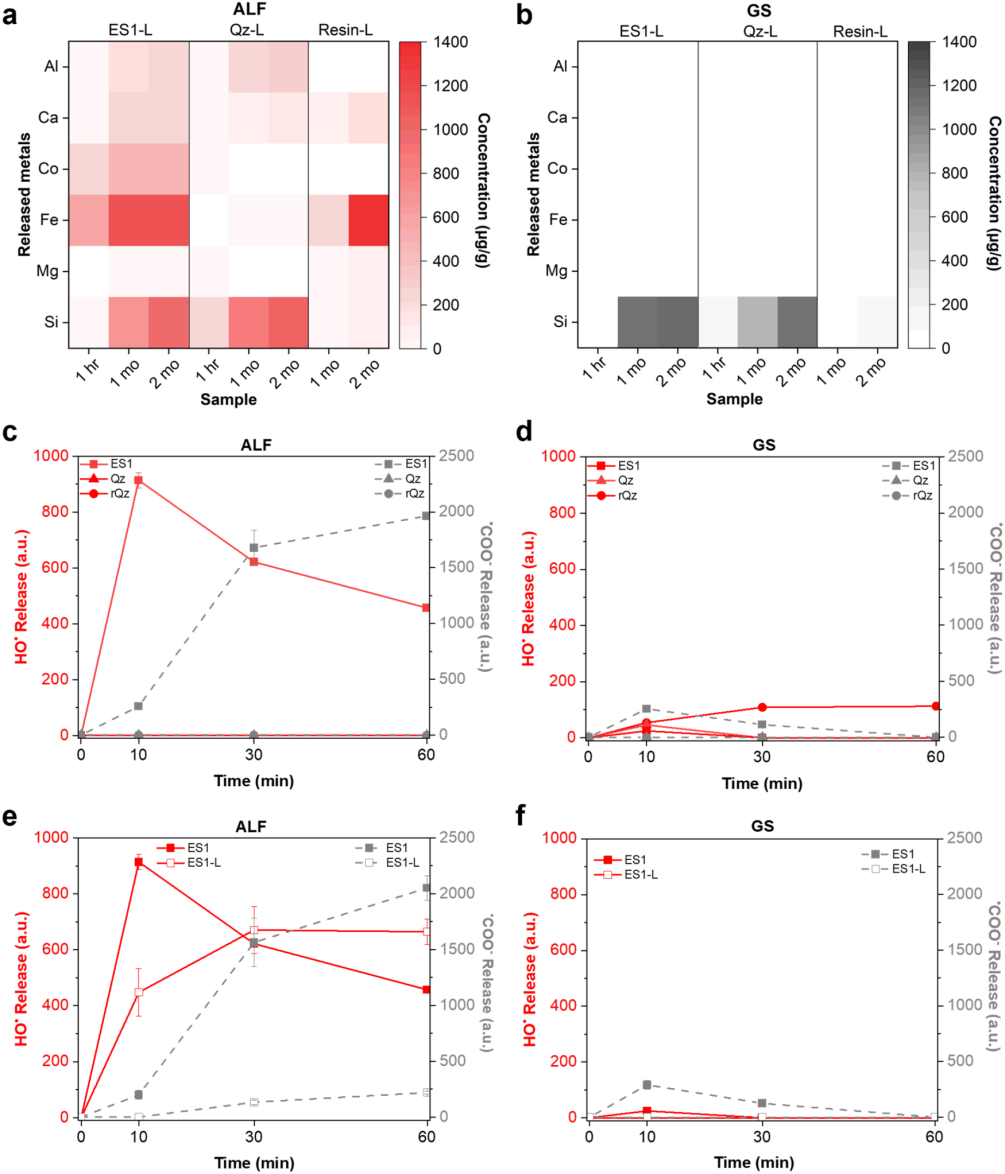
Metal ion leaching and free radical generation from ES dust in simulated body fluids. (a, b) Metal concentrations (μg/g) measured by ICP-OES in the leachates of ES1 (ES1-L), pure quartz (Qz-L), or resin (resin-L) incubated in ALF (a) or GS (b) for 1 h,1 and 2 months. Data are expressed as mean ± SD of two independent experiments. (c, d) Kinetics of formation of hydroxyl (^•^OH) and carboxyl (^•^COO^–^) radicals from ES1, pure quartz (Qz), or a reference quartz (rQz) incubated in ALF (c) or GS (d) with DMPO as trapping agent and hydrogen peroxide or sodium formate as target molecules. The amount of released radicals was measured after 10, 30, and 60 min of incubation and is reported as arbitrary units ± SEM of two independent experiments. (e, f) Kinetics of formation of hydroxyl (^•^OH) and carboxyl (^•^COO^–^) radicals generated by ES1 particles or ES1 leachates (ES1-L). To obtain ES1 leachates (ES1-L), ES1 was preincubated for 60 min in ALF (e) or GS (f). The particle-free leachate was added to DMPO and hydrogen peroxide or sodium formate and radicals monitored for 10, 30, and 60 min. Data are mean ± SEM of two independent experiments.

Incubation in the GS promoted a rather low release of metals for both ES1 (Co 9.2 ± 0.1 μg/g; mean ± SD) and Qz (Al 5.3 ± 0.9 μg/g and Co 0.64 ± 0.03 μg/g; mean ± SD) after 1h (Figure 3b and Table S5). No release of metals was revealed at higher time points, except a time-dependent increase of Si for all samples investigated, similarly to what observed in ALF. These data suggest that a minor amount of amorphous siliceous material may dissolve over time in simulated body fluids.

Overall, the analysis of ES leachates confirmed previous findings in ALF regarding the occurrence of Fe and Al.^44^ Moreover, the comparison between ALF and GS leachates indicated that the leaching of inorganic species is significantly influenced by the composition of the medium, particularly its pH and the presence of chelating agents, such as citrate, which is present at high concentration in ALF. Speciation studies (Tables S6–8 and Figure S5) revealed that no insoluble metal species formed in either ALF or GS as saturation conditions for the possible solid species (Table S7) were not reached. Specifically, in the leachate from ES1 in ALF (pH 4.5) (Figure S5), the cations (Ca^2+^, Mg^2+^, Al^3+^, Fe^3+^, and Co^2+^) were predominantly present as citrate complexes, often accounting for more than 90% of each element speciation. Sodium existed mainly as free ion, while silicon was almost entirely present as Si­(OH)_4_(aq). Instead, in GS (pH 7.4), alkaline and alkaline earth metals were mostly found as free cations and Fe^3+^ predominantly interacted with PO_4_^3–^ (Figure S5). The same difference in metal speciation might happen in different tissue or cell compartments where the chemical environment might change. Speciation calculations for Qz and resin leachates in both media yielded element distributions comparable to those observed for ES1. Because transition metal ions, including Fe and Co, might catalyze oxidative stress reactions, the capacity of the ES dust to induce hydroxyl (^•^OH) and carboxyl (^•^COO^–^) radicals was assessed both in ALF (Figure 3c) and in GS (Figure 3d). Among ROS, the highly oxidant ^•^OH is one of the most abundant specie involved in cell damage by different types of materials because of its strong oxidation potential (2.4 V for the ^•^OH/H_2_O redox couple) and its ability to oxidize a wide range of organic molecules, including DNA, proteins, and phospholipids.^60^ The formation of ^•^COO^–^ from sodium formate is representative of the homolytic cleavage of labile C–H bonds in biomolecules.^61,62^ ES1, Qz and rQz were incubated with hydrogen peroxide (H_2_O_2_) or sodium formate (HCOO^–^ Na^+^) to assess ^•^OH and carboxyl ^•^COO^–^ radical formation, respectively. The spin trapping agent DMPO forms stable adducts with the radicals, i.e., [DMPO–OH]˙ or [DMPO–COO]˙^–^, that could be detected by EPR spectroscopy.^61^ Representative EPR spectra of the ^•^OH and ^•^COO^–^ adducts recorded after 10 min of incubation of ES1, Qz, and the rQz in ALF or GS are reported in Figure S6. Quantitative data, obtained by spectra integration of a three-point kinetics at 10, 30, and 60 min, are presented in Figure 3c,d.

In ALF (Figure 3c), ES1 induced a strong formation of ^•^OH and ^•^COO^–^ radicals compared to the pure quartz samples (Qz and rQz), confirming previous findings that demonstrated the high capacity of ES dust to generate free radicals.^22^ A quantitative comparison of the signal intensities between the two radical species generated by ES1 is not meaningful, as they correspond to different reactive species characterized by distinct reactivity and stability of their respective DMPO adducts.^61^ Notably, the steep kinetics followed by a decay phase observed for the formation of [DMPO–OH]˙ from ES1 particles suggests a fast formation of highly reactive ^•^OH radicals, followed by a self-quenching process resulting from their tendency to recombine either with each other or with the particle surface.^63^ In contrast, the formation of [DMPO–COO]˙̅ followed a slower kinetics, attaining its maximum after 60 min of incubation. This behavior is consistent with the lower reactivity and longer lifetime of ^•^COO^–^ compared to ^•^OH radicals.^64^

In GS (Figure 3d), all tested particles induced a slight increase in ^•^OH generation after 10 min of incubation. However, the radical intensity observed in GS was much lower than that of ALF for ES1. This difference may be attributed to the lower concentration of transition metals leached during incubation in GS with respect to ALF, particularly Fe and Co at 1 h incubation time (Figure 3a,b). Notably, the spectrum of 5,5-dimethylpyrroline-(2)-oxy(1) (DMPOX) was observed only for ES1 incubated in GS for 30 and 60 min (Figure S7). This species has been previously reported in the presence of Co, suggesting the production of strong oxidants.^65^ According to Rosen and Rauckman,^66^ the DMPOX signal represents indirect evidence for peroxyl radical (ROO^·^) generation, following its trapping by DMPO. In this context, the detection of DMPOX may be due to the oxidative activity of Co, which is released from ES1 after 1 h incubation (Table S5). In GS, only ES1 slightly induced ^•^COO^–^ formation, while Qz and rQz were inactive (Figure 3d).

We further explored the mechanisms of radical formation by ES1 to determine whether they arise from the metals leached into solution or from defects on the particle surface. To this aim, ES1 dust was preincubated in ALF or GS for 60 min. Then, the dust was removed and H_2_O_2_ or sodium formate added to the dust-free leachates (ES1-L), and spectra recorded up to 60 min (Figure 3e,f). Hydroxyl radical (^•^OH) yield induced by ALF leachates of ES1 (Figure 3e) increased in a time-dependent manner for the first 30 min and then reached a plateau. This suggested that transition metals released in the ALF leachates of ES1 (Figure 3a) could rapidly and stably induce radical formation. The kinetics of ^•^OH formation from the ES1 leachate differed from that of ES1 particles. While the ^•^OH signal associated with the particles decreased after 30 min, the leachate – lacking particulate matter – displayed a continuous increase followed by a plateau rather than a decay. This supports the interpretation that the decrease of ^•^OH in the presence of ES1 particles is due to a self-quenching process, likely resulting from the high reactivity of ^•^OH radicals and their tendency to recombine with reactive sites on the particle surface.^64^ Regarding the ^•^COO^–^ yield in ALF (Figure 3e), the particulate-mediated reactivity was predominant with respect to the leachate, possibly indicating that particle surface defects or surface-bound metals mediate the rupture of the C–H bond of formate.

Leachates from ES1 in GS (Figure 3f) showed a lower induction of radical formation, both ^•^OH and ^•^COO^–^, compared to ES1 particles, which already exhibited very weak spectral signals. Furthermore, the overall release in GS was markedly lower than that observed in ALF leachates, in agreement with the trends observed in the particulate studies.

Overall, these data suggested that incubation of ES1 in ALF induced metal release, in particular Fe and Co, possibly bound to citrate, that catalyze the formation of ^•^OH through Fenton or Fenton-like reactions. ES1 in its particulate form strongly induced ^•^COO^–^ release in ALF, suggesting that particle surface defects are also important in the contribution of ES oxidative stress. Di Benedetto and co-workers^24^ highlighted the generation of unique stable surface radicals associated to the cleavage of the Si–O bonds of crystalline silica and the role of the resin in protecting these surface-bound radicals from annihilation. These stable particle surface radicals may play a role in particle-related ^•^COO^–^ release. In general, the free radical yield from ES1 particles or leachates was higher in ALF than in GS, and this was related to the negligible release of metals observed for the GS leachates.

## 4 Conclusions

In summary, this study clarifies the role of particle surface features in the mechanisms underlying the toxicity of high-crystalline-silica engineered stone (ES), highlighting two primary structural drivers of toxicity. The first mechanism involves surface silanol groups on crystalline silica, particularly nearly free silanols (NFS). These reactive sites are initially masked by the resin coating but may become exposed upon prolonged interaction with physiological-like environments, such as the acidic fluid within lysosomes, as indicated by results from artificial lysosomal fluid (ALF) incubation and thermal oxidation treatment. Once nearly free silanols (NFS) are exposed, they can directly interact with cellular membranes, leading to membrane destabilization and lysis.^39,41^

The second mechanism is associated with the generation of reactive radical species (ROS and R^•^), including hydroxyl, carboxyl, and peroxyl radicals. These reactions are catalyzed by redox-active species, such as Fe and Co, either naturally present in the slab raw materials or introduced during the slab manufacturing process. This radical-driven pathway might be responsible for an early toxic effect boosting cellular oxidative stress and contributing to lung injury.

In addition to NFS and metal-mediated oxidative stress, other factors may contribute to the toxicity of ES dust. For instance, previous work has identified crystalline silica nanoparticles (in the sub-100 nm fraction) and other minerals in aerosols released during the grinding of ES.^67^ Moreover, the resin itself may play a role, as dry-cut ES slabs has been shown to release volatile organic compounds (VOCs) that are irritant to the respiratory tract.^68^ It is also possible that resin residues on particle surfaces could interact with cells, for example through radical generation or other mechanisms of toxicity, similar to those described for micro- and nanoplastics.^69^

While this study provides important insights into the mechanisms of action of ES dust, it has limitations. Chemical characterization of the diamond saw blade used for dust generation would help to determine whether the detected impurities originated from the ES formulation or were introduced by the cutting tool. In this regard, the investigation of the chemical variability of the ES dust that may arise from the use of dyes and pigments in commercial ES slabs should be extended to consider additional redox activity induced by transition metal ions and particles. The use of two lung simulants (ALF and GS) offers a simplified model of the *in vivo* environment, which may not fully capture the complexity of biological responses.

Further *in vitro* and *in vivo* studies, particularly those evaluating the effects of removing the organic resin layer, are urgently needed to understand the peculiar interaction of ES dust with cells and tissues. Although ES-induced lung pathology shares similarities with traditional silicosis – namely, inflammation and fibrosis – the underlying molecular mechanisms may differ due to the unique physicochemical characteristics of ES, such as resin coatings and additives. Continued research is crucial to fully elucidate these mechanisms and to inform preventive strategies in occupational health settings.

## Supplementary Material



## Data Availability

The data underlying this study are available in the published article and its Supporting Information.

## Notes

Methods for determination of crystallinity, particle morphology, size distribution, elemental composition, specific surface area, and speciation of dust leachates in ALF and GS. Tables and figures relative to dust characterization, dust leachates in ALF and GS, membranolytic activity, and EPR spectra. Supporting references (PDF)

## #

Department of Chemistry and Biochemistry, Mendel University in Brno, Brno 61300, Czech Republic
