# Supplementary material for: The Combined Role of Silanols and Oxidative Stress in Determining Engineered Stone Dust Toxicity
Source: ACS Org Inorg Au. 2025 Nov 5;6(1):76–87. doi: 10.1021/acsorginorgau.5c00089 (PMC12879171; doi:10.1021/acsorginorgau.5c00089)
Supplement: Supplementary file 1 [file gg5c00089_si_001.pdf]

Supporting Information

The Combined Role of Silanols and Oxidative Stress in Determining Engineered Stone Dust Toxicity

Cristina Pavan[a,b,c], Marianna Fimiani[a,b], Stefania Cananà[a,b], Aleandro Diana[a], Matteo Marafante[a], Stefano Bertinetti[a], Guillermo Escolano-Casado[a†], Lorenzo Mino[a], Dino Pisaniello[d], Riccardo Leinardi[a,b,c], Maura Tomatis[b,e], Francesco Turci[a,b]*

[a] Department of Chemistry, University of Turin, Turin, 10125, Italy

[b] "G. Scansetti" Interdepartmental Centre for Studies on Asbestos and Other Toxic Particulates, University of Turin, Turin, 10125, Italy

[c] Louvain Centre for Toxicology and Applied Pharmacology, Institut de Recherche Expérimentale et Clinique (IREC), Université catholique de Louvain, Brussels, 1200, Belgium

[d] Adelaide Exposure Science and Health, School of Public Health, University of Adelaide, Adelaide, 5005, Australia

[e] Department of Veterinary Sciences, University of Turin, Grugliasco, 1095, Italy

[†]Present address: Department of Chemistry and Biochemistry, Mendel University in Brno, Brno, 61300, Czech Republic.

*Corresponding author: francesco.turci@unito.it

Table of Contents:

1. Supplemental Methods 3

   1.1 Crystallographic analysis of ES dusts 3

   1.2 Particle morphology and size distribution 3

   1.3 Elemental composition of the dust 4

   1.4 Specific Surface Area (SSA) with Kr-BET 4

   1.5 Speciation of ES dust leachates in ALF and GS 4

2. Supplemental Tables 6

   Table S1. Mineralogical and elemental composition of the tested particles. 6

   Table S2. Specific surface area (SSA) of pristine, incubated, and thermally treated ES and quartz samples determined by the Kr-BET method according to Supplementary methods. 7

   Table S3. Loss of resin from ES samples before and after incubation in ALF for 2 months evaluated by TGA analysis. 7

   Table S4. Release of metals (μg/g ± SD) from ES1, Qz and resin incubated in ALF for 1h, 1 and 2 months measured by ICP-OES. Elements below the Limit of Detection are expressed as <LOD. 8

   Table S5. Release of metals (μg/g ± SD) from ES1, Qz and resin incubated in GS for 1h, 1 and 2 months measured by ICP-OES. Elements below the Limit of Detection are expressed as <LOD. 8

   Table S6. Stability constants logβ of the considered species at 25 °C and various ionic strength *I*. Charges of the species were omitted for simplicity. 9

   Table S7. Solubility product log*Ksp* of the considered species at 25 °C and various ionic strength *I*. Charges of the species were omitted for simplicity. 12

   Table S8. Concentration of components in the leachates of ES1, Qz, and Resin in ALF and GS. 12

3. Supplemental Figures 13

   Fig. S1. Micromorphology of ES1 (a, b) and Qz (c, d) obtained by SEM microscopy 13

   Fig. S2. Particle size of ES1, Qz and reference quartz (rQz) 14

   Fig. S3. Thermogram (solid red line) and derivative curve (dotted black line) of pure quartz (Qz) obtained by TGA analysis in artificial air. 15

   Fig. S4. Membranolytic activity (hemolysis, %) at increasing particle concentration (mg/ml) of pristine ES2 or ES4 dust, ... 15

   Fig. S5. Element distribution among the different chemical species, expressed as percentage, for ES1 leachate in ALF at pH 4.5 (a) and in GS at pH 7.4 (b). 16

   Fig. S6. EPR spectra of the $[DMPO\text{-}OH]^{\cdot}$ or $[DMPO\text{-}COO]^{\cdot-}$ adducts recorded after 10 min of incubation of ES1, pure quartz (Qz), or a reference quartz (rQz) with ALF or GS, DMPO and H2O2 or sodium formate 17

   Fig. S7. EPR spectra recorded after 10, 30 and 60 min of ES1 incubation in GS, DMPO and H2O2. The spectra reported represent a single measurement, selected as representative of multiple measurements. .. 17

4. Supplemental references 18

1. Supplemental Methods

1.1 Crystallographic analysis of ES dusts

Crystallographic analysis was carried out on dry samples by X Ray Diffraction (XRD), using a PW3050/60 X'Pert Powder X-Ray Diffractometer (Malvern Panalytical), in a spinner configuration to eliminate the preferential orientation of the powder (1 rotation/s). Diffractograms were collected between 5°-90° (2θ), using Cu-Kα radiation at 45 kV and 40 mA. Crystal phases and crystal quality were identified and evaluated by Rietveld data analysis, using the Materials Analysis Using Diffraction (MAUD) software. Quantitative analysis of the amorphous phase was obtained employing the method of the internal standard (20 wt.% of Al_2O_3), mixing the dry powder in an agate mortar.

1.2 Particle morphology and size distribution

Micromorphology and size analysis was assessed by Scanning Electron Microscopy (SEM) using a Zeiss EVO MA10 microscope (Carl Zeiss), equipped with a tungsten source. Each particle (1 mg) was suspended in 10 ml of ultrapure water. The suspension was sonicated (2 min, 50% amplitude, power 25 W) on ice, using a Sonopuls HD3200 ultrasound probe (Bandelin). To prevent sedimentation, the dispersion was magnetic stirred for 20 min, at room temperature. To improve disaggregation of particles, the pH of the suspension was changed to ca. 8, by adding 0.01M NaOH and 0.1% Triton X-100. Using a vacuum filtration system, a proper amount of the suspension was transferred on a nitrocellulose membrane (2.5 cm diameter, 0.2 μm porosity) to deposit ca. 0.1 mg of the sample on the membrane (Sartorius). The membrane was then mounted on an aluminum sample holder using a graphite tape and was coated with a 17 nm carbon layer. Images for micromorphology were taken at 15 kV and different magnifications. For size analysis, images were taken at a specific magnification (1,000×), at a resolution of 2048x2048 pixels, that allowed to detect particles ranging from 0.14 μm to tens of microns. To obtain statistically significant results, each membrane was virtually divided into four quarters (NE, SE, SW, NW), and in each quarter, a randomly identified

area (0.5x0.5 mm) was automatically analyzed using AZtecFeature suite (Oxford Instruments). More than 3,000 particles were detected per sample.

Size distribution was also assessed by Flow Particle Image Analysis (FPIA) on particles suspended in milliQ water (0.5 mg/ml) and sonicated (2 min, 50% amplitude, power 25 W) on ice, using a Sonopuls HD3200 ultrasound probe (Bandelin). The size distribution of particles was then measured using a FPIA-3000S (Malvern Instruments). Particle dispersions were injected (ca. 5 ml) into the measurement cell and stirring was applied (360 rpm) to avoid particle sedimentation. Particle images were captured using stroboscopic illumination and a charge-coupled device camera. Data were processed by the Sysmex FPIA software (version 00-13). The detection range was 0.8 to 160 µm. The average diameter expressed as Equivalent Circle Diameter (ECD) was reported.

1.3 Elemental composition of the dust

The elemental composition of the dust was carried out by Energy Dispersive X Ray Spectroscopy (EDS) on the membranes prepared for particle morphology by using a Zeiss EVO MA10 microscope coupled with an INCA Energy 250 EDS system with X-Act SDD detector (Oxford Instruments). Data were collected and processed using Aztec Feature suite (Oxford Instruments). The EDS spectra were automatically acquired for each particle detected by the software. More than 3,000 particles were detected per sample and sorted out on in classes based on the EDS spectra.

1.4 Specific Surface Area (SSA) with Kr-BET

The SSA of the particles was evaluated by the Brunauer, Emmett, and Teller (BET) method, using an ASAP 2020 porosimeter (Micromeritics) and Kr adsorption at -196 °C. Samples were degassed at room temperature for 24h before the analysis. BET surface area was then calculated over the range $P/P_0 = 0.06 - 0.20$ (9 points).

1.5 Speciation of ES dust leachates in ALF and GS

The distribution of species in the leachates obtained from ALF and GS was computed using the PyES software.[1] The speciation model included several cations - H^+, Na^+, K^+, Ca^{2+}, Mg^{2+}, Al^{3+}, Si^{4+} (as

$Si(OH)_4$), Fe^{3+} and Co^{2+} - and anions - Cl^{-}, PO_4^{3-}, SO_4^{2-}, CO_3^{2-}, acetate, citrate, tartrate, pyruvate, lactate and glycine. Stability constants of the species were collected from Brown and Ekberg 2016,[2] Thoenen et al. 2014,[3] Hummel and Thoenen 2023,[4] Baes and Mesmer 1977,[5] and The IUPAC Stability Constants Database.[6] Both soluble and solid species that could potentially form in solution were considered in the speciation model. The selected stability constants, originally reported at various ionic strengths, were corrected to the ionic strength of the solution using an extended version of the Debye-Hückel equation.[7] The soluble species and their stability constants are reported in Table S6, while the solid species and their solubility products are listed in Table S7. The total concentrations of components in the leachates were calculated considering the composition of the ALF and GS, and the soluble fraction of ES1, Qz, and resin. These concentrations are reported in Table S8.

2. Supplemental Tables

Table S1. Mineralogical and elemental composition of the tested particles.

Particles	Mineralogical composition[a] (wt.%)	Transition metals[b] (atomic wt.%)
**ES1**	Quartz (84.2), albite (9.0), amorphous (6.8)	Al (1.22), Ti (2.01), Fe (0.10), Zr (0.58)
**ES2**	Quartz (23), cristobalite (43), albite (7.2), rutile (0.5), other (26.3)	Fe (0.01), Al (0.72), Ti (0.19), Mn (<0.01), Cr (<0.01), Cu (<0.01), Ni (<0.01), Co (<0.01), Pb (<0.01)
**ES3**	Quartz (90), cristobalite (0.3), hematite (0.1), other (9.6)	Fe (<0.01), Al (0.26), Ti (0.29), Mn (<0.01), Cr (<0.01), Cu (<0.01), Ni (<0.01), Co (<0.01), Pb (<0.01)
**ES4**	Quartz (99), other (1)	Fe (0.09), Al (0.23), Ti (0.08), Mn (<0.01), Cr (<0.01), Cu (<0.01), Ni (<0.01), Co (0.01), Pb (<0.01)
**ES5**	Quartz (99), magnetite (0.01), other (0.09)	Fe (0.06), Al (0.14), Ti (0.02), Mn (<0.01), Cr (<0.01), Cu (<0.01), Ni (<0.01), Co (0.01), Pb (<0.01)
**ES6**	Quartz (99), albite (0.03), rutile (0.9), other (0.07)	Fe (0.22), Al (0.25), Ti (0.01), Mn (<0.01), Cr (<0.01), Cu (<0.01), Ni (<0.01), Co (<0.01), Pb (<0.01)
**ES7**	Quartz (76), cristobalite (23), rutile (0.6), other (0.4)	Fe (<0.01), Al (0.20), Ti (0.77), Mn (< 0.01), Cr (< 0.01), Cu (< 0.01), Ni (< 0.01), Co (0.01), Pb (< 0.01)
**ES8**	Quartz (99), other (1)	Fe (0.02), Al (0.40), Ti (0.54), Mn (<0.01), Cr (<0.01), Cu (<0.01), Ni (<0.01), Co (<0.01), Pb (<0.01)
**ES9**	Quartz (96), rutile (4)	Fe (0.02), Al (0.13), Ti (0.15), Mn (<0.01), Cr (<0.01), Cu (<0.01), Ni (<0.01), Co (0.01), Pb (<0.01)
**Qz**	Quartz (83.2), albite (16.8), amorphous (0.0)	Al (1.87), Ti (1.67), Fe (0.04), Zr (2.11)
**rQz**	Quartz (98.8)	Al (1.2)

[a] ES1 and Qz were evaluated by XRD analysis and Rietveld refinement according to Supplemental methods; the data for ES2-ES9 represent wt.% of the total crystalline content, as determined by XRD analysis in the study by Kumarasamy *et al.*;[8]

[b] ES1 and Qz were evaluated by EDS analysis according to Supplemental methods; the data for ES2-ES9 represent wt.% of the total crystalline content, as determined by X ray fluorescence (XRF) analysis in the study by Kumarasamy *et al.*[8]

Table S2. Specific surface area (SSA) of pristine, incubated, and thermally treated ES and quartz samples determined by the Kr-BET method according to Supplementary methods.

Particles	SSA (m^2/g) (SD ± 5%)
**ES1**	2.0
**ES1+ALF**	1.4
**ES1-400°C**	2.8
**ES2**	1.6
**ES4**	1.5
**Qz**	1.7
**rQz**	5.0

Table S3. Loss of resin from ES samples before and after incubation in ALF for 2 months evaluated by TGA analysis.

ES samples	Resin loss before ALF incubation (wt.%)	Resin loss after ALF incubation (wt.%)	Resin loss on the total resin (%)
**ES1**	11.7	9.0	23.1
**ES2**[a]	14.3	14.1	1.4
**ES3**[a]	12.6	10.5	16.7
**ES4**[a]	9.7	9.6	1.0
**ES5**[a]	14.6	13.5	7.5
**ES6**[a]	12.7	11.9	6.3
**ES7**[a]	8.9	8.0	10.1
**ES8**[a]	11.0	9.1	17.3
**ES9**[a]	10.8	9.8	9.3
**Qz**[b]	0.3	0.1	-
**Resin**	99.4	-	-

[a] Data for ES2-ES9 refer to the study by Kumarasamy *et al.*[8]
[b] No resin loss expected

Table S4. Release of metals (µg/g ± SD) from ES1, Qz and resin incubated in ALF for 1h, 1 and 2 months measured by ICP-OES. Elements below the Limit of Detection are expressed as <LOD.

	**ES1**			**Qz**			**Resin**	
	**1 hr**	**1 mo**	**2 mo**	**1 hr**	**1 mo**	**2 mo**	**1 mo**	**2 mo**
**Al**	18.7 ± 0.7	190.46 ± 0.02	264.91 ± 0.01	18.952 ± 0.003	257.6 ± 68.2	312.6 ± 63.7	<LOD	<LOD
**Ca**	13.0 ± 1.7	212.9 ± 0.1	219.17 ± 0.02	6.3 ± 3.5	96.2 ± 37.6	128.6 ± 0.3	96.54 ± 0.01	188.74 ± 0.04
**Co**	214.7 ± 5.1	461.46 ± 0.02	453.75 ± 0.01	0.73 ± 0.01	<LOD	<LOD	<LOD	<LOD
**Fe**	555.1 ± 8.0	1157.84 ± 0.01	1143.83 ± 0.02	<LOD	8.2 ± 6.9	13.7 ± 6.0	213.334 ± 0.001	1340.43 ± 0.04
**Mg**	<LOD	35.07 ± 0.02	28.23 ± 0.01	4.1 ± 2.4	<LOD	<LOD	28.95 ± 0.02	51.54 ± 0.01
**Si**	10.0 ± 1.6	719.46 ± 0.03	989.93 ± 0.02	212.2 ± 7.2	828.2 ± 108.4	1034.7 ± 51.2	13.86 ± 0.01	61.082 ± 0.005

Table S5. Release of metals (µg/g ± SD) from ES1, Qz and resin incubated in GS for 1h, 1 and 2 months measured by ICP-OES. Elements below the Limit of Detection are expressed as <LOD.

	**ES1**			**Qz**			**Resin**	
	**1 hr**	**1 mo**	**2 mo**	**1 hr**	**1 mo**	**2 mo**	**1 mo**	**2 mo**
**Al**	0.72 ± 0.02	<LOD	<LOD	5.3 ± 0.9	<LOD	<LOD	<LOD	<LOD
**Ca**	<LOD	<LOD	<LOD	<LOD	<LOD	<LOD	<LOD	<LOD
**Co**	9.2 ± 0.1	<LOD	<LOD	0.64 ± 0.03	<LOD	<LOD	<LOD	<LOD
**Fe**	<LOD	<LOD	<LOD	<LOD	<LOD	<LOD	<LOD	<LOD
**Mg**	<LOD	<LOD	<LOD	10.0 ± 4.8	<LOD	<LOD	<LOD	<LOD
**Si**	34.4 ± 1.6	1109.8 ± 90.7	1154.3 ± 7.5	178.9 ± 5.5	782.2 ± 77.8	1121.4 ± 54.6	59.75 ± 0.01	108.39 ± 0.01

Table S6. Stability constants log*β* of the considered species at 25 °C and various ionic strength *I*. Charges of the species were omitted for simplicity.

**H^+**			**Na^+**			**K^+**		
**Species**	**log*β***	***I*** [mol·L^{-1}]	**Species**	**log*β***	***I*** [mol·L^{-1}]	**Species**	**log*β***	***I*** [mol·L^{-1}]
OH	-13.97	0.00	(Na)(OH)	-14.4	0.00	(K)(OH)	-14.5	0.00
$(PO_4)(H)$	12.35	0.00	$(Na)(PO_4)$	1.43	0.00	$(K)(PO_4)$	1.37	0.00
$(PO_4)(H)_2$	19.55	0.00	$(Na)(PO_4)(H)$	13.4	0.00	$(K)(PO_4)(H)$	13.21	0.00
$(PO_4)(H)_3$	21.698	0.00	$(Na)(PO_4)(H)_2$	19.81	0.00	$(K)(PO_4)(H)_2$	19.79	0.00
$(SO_4)(H)$	1.987	0.00	$(Na)2(PO_4)$	2.59	0.00	$(K)_2(PO_4)$	2.2	0.00
(Ac)(H)	4.67	0.10	$(Na)2(PO_4)(H)$	13.32	0.00	$(K)_2(PO_4)(H)$	13.44	0.00
$(CO_3)(H)$	10.336	0.00	$(Na)(SO_4)$	0.83	0.00	$(K)(SO_4)$	0.91	0.00
$(CO_3)(H)_2$	16.69	0.00	(Na)(Ac)	-0.07	0.00	(K)(Ac)	-0.43	0.25
(Cit)(H)	5.58	0.20	$(Na)(CO_3)$	1.29	0.00	(K)(Cit)	1.42	0.00
$(Cit)(H)_2$	9.85	0.20	(Na)(Cit)	1.54	0.00	(K)(Cit)(H)	7.13	0.00
$(Cit)(H)_3$	12.76	0.20	(Na)(Cit)(H)	7.33	0.00	$(K)(Cit)(H)_2$	11.3	0.00
(Tar)(H)	4.25	0.10	$(Na)(Cit)(H)_2$	11.4	0.00	$(K)_2(Cit)_2(H)$	7	0.00
$(Tar)(H)_2$	7.5	0.10	$(Na)_2(Cit)(H)$	7	0.00	$(K)_2(Cit)_2$	1.93	0.00
(Pyr)(H)	2.47	0.00	$(Na)_2(Cit)$	2.38	0.00	(K)(Gly)(H)	8.78	0.25
(Lac)(H)	3.653	0.15	(Na)(Tar)	0.58	0.25	(K)(Cl)	-0.51	0.00
(Gly)(H)	9.6	0.10	(Na)(Tar)(H)	4.05	0.25			
$(Gly)(H)_2$	11.93	0.10	(Na)(Gly)	-0.4	0.50			
			(Na)(Gly)(H)	9.1	0.50			
			(Na)(Cl)	-0.6	0.00			

Table S6. *–continued*

Ca^{2+}			Mg^{2+}			Al^{3+}		
**Species**	**log*β***	***I*** [mol·L^{-1}]	**Species**	**log*β***	***I*** [mol·L^{-1}]	**Species**	**log*β***	***I*** [mol·L^{-1}]
(Ca)(OH)	-12.57	0.00	(Mg)(OH)	-11.7	0.00	(Al)(OH)	-4.98	0.00
(Ca)(PO_4)	0.96	0.00	$(Mg)_4(OH)_4$	-39.71	0.00	$(Al)(OH)_2$	-10.63	0.00
$(Ca)(PO_4)_2$	1.69	0.00	(Mg)(PO_4)(H)	13.8	0.25	$(Al)(OH)_3$	-15.66	0.00
(Ca)(SO_4)	2.35	0.00	(Mg)(SO_4)	2.22	0.00	$(Al)(OH)_4$	-22.91	0.00
(Ca)(Ac)	1.12	0.00	(Mg)(Ac)	1.7	0.00	$(Al)_2(OH)_2$	-7.62	0.00
(Ca)(Cit)	2.71	0.50	(Mg)(CO_3)	2.98	0.00	$(Al)_3(OH)_4$	-13.9	0.00
(Ca)(Cit)(H)	6.7	0.50	(Mg)(Cit)	4.71	0.00	$(Al)_{13}(OH)_{32}$	-100.03	0.00
(Ca)(Tar)	2.1	0.25	(Mg)(Cit)(H)	8.84	0.00	(Al)(PO_4)(H)	17.6	0.20
(Ca)(Tar)(H)	5.02	0.25	$(Mg)(Cit)(H)_2$	12.2	0.00	(Al)(PO_4)(OH)	8.37	0.20
(Ca)(Pyr)	2.23	1.00	(Mg)(Tar)	0.35	0.00	$(Al)(PO_4)(H)_2$	19.65	0.20
(Ca)(Lac)	0.92	0.50	(Mg)(Pyr)	2.05	1.00	$(Al)_2(PO_4)$	17.42	0.20
$(Ca)(Lac)_2$	1.62	0.50	(Mg)(Lac)	0.93	0.50	(Al)(SO_4)	3.84	0.00
(Ca)(Gly)	2.86	0.10	(Mg)(Gly)	3.09	0.10	$(Al)(SO_4)_2$	5.58	0.00
(Ca)(Cl)	0.42	0.00	(Mg)(Cl)	0.49	0.00	(Al)(Ac)	2.02	0.10
						$(Al)(Ac)_2$	3.5	0.10
						(Al)(Cit)	7.85	0.20
						$(Al)(Cit)_2$	12.73	0.20
						(Al)(Cit)(H)	10.18	0.20
						$(Al)(Cit)(OH)_2$	-1.77	0.20
						(Al)(Cit)(OH)	4.27	0.20
						$(Al)(Cit)_2(OH)$	7.81	0.20
						$(Al)(Tar)(OH)_2$	-7.8	0.50
						(Al)(Lac)	2.36	0.60
						$(Al)(Lac)_2$	4.42	0.60
						$(Al)(Lac)_3$	5.79	0.60
						(Al)(Gly)	5.92	0.10
						$(Al)(Gly)_2$	10.27	0.10
						(Al)(Cl)	1.73	0.20
						$(Al)(Cl)_2$	2.2	0.20

Table S6. *–continued*

$Si(OH)_4$			Fe^{3+}			Co^{2+}		
**Species**	**log*β***	***I* [mol·L^{-1}]**	**Species**	**log*β***	***I* [mol·L^{-1}]**	**Species**	**log*β***	***I* [mol·L^{-1}]**
$(Si(OH)_4)(OH)$	-9.81	0.00	$(Fe)(OH)$	-2.2	0.00	$(Co)(OH)$	-9.61	0.00
$(Si(OH)_4)(OH)_2$	-23.14	0.00	$(Fe)(OH)_2$	-5.71	0.00	$(Co)(OH)_2$	-19.77	0.00
$(Si(OH)_4)_4(OH)_2$	-13.44	0.00	$(Fe)(OH)_3$	-12.26	0.00	$(Co)(OH)_3$	-32.01	0.00
$(Si(OH)_4)_4(OH)_4$	-36.3	0.00	$(Fe)(OH)_4$	-21.6	0.00	$(Co)(OH)_4$	-46.3	0.00
$(Si(OH)_4)(SO_4)$	0.544	0.00	$(Fe)_2(OH)_2$	-2.91	0.00	$(Co)_2(OH)$	-11.2	0.00
			$(Fe)_3(OH)_4$	-6.3	0.00	$(Co)_4(OH)_4$	-30.53	0.00
			$(Fe)(PO_4)(H)_2$	20.71	0.15	$(Co)(PO_4)$	0.96	0.00
			$(Fe)(PO_4)(H)$	19.89	0.15	$(Co)(PO_4)_2$	1.89	0.00
			$(Fe)(PO4)_2$	35.66	0.15	$(Co)(PO_4)(H)$	2.22	0.10
			$(Fe)(PO4)_2(OH)_2$	32.16	0.15	$(Co)(SO_4)$	2.25	0.00
			$(Fe)(SO_4)$	3.82	0.00	$(Na)(CO_3)$	1.29	0.00
			$(Fe)(SO_4)_2$	5.75	0.00	$(Mg)(CO_3)$	2.98	0.00
			$(Fe)(SO_4)(H)$	3.68	0.00	$(Co)(Ac)$	1.59	0.10
			$(Fe)(Ac)$	3.2	1.00	$(Co)(Ac)_2$	2.21	0.10
			$(Fe)(Ac)_2$	6.3	1.00	$(CO_3)(H)$	10.336	0.00
			$(Fe)(Ac)_3$	8.2	1.00	$(CO_3)(H)_2$	16.69	0.00
			$(Fe)(CO_3)_2$	7.4	0.20	$(Fe)(CO_3)_2$	7.4	0.20
			$(Fe)(Cit)$	9.5	1.00	$(Co)(CO_3)$	4.7	0.10
			$(Fe)(Cit)_2$	15.3	1.00	$(Co)(Cit)$	4.63	0.20
			$(Fe)(Cit)(OH)$	7.31	1.00	$(Co)(Cit)_2$	7.01	0.20
			$(Fe)(Cit)_2(H)$	10.46	1.00	$(Co)(Cit)(H)$	8.28	0.20
			$(Fe)(Cit)_2(OH)$	19.12	1.00	$(Co)(Cit)_2(OH)_2$	-3.52	0.20
			$(Fe)(Tar)$	6.23	0.50	$(Co)(Tar)$	5.22	0.10
			$(Fe)_2(Tar)_2(OH)_3$	9.25	0.50	$(Co)(Pyr)$	0.6	2.00
			$(Fe)_3(Tar)_3(OH)_6$	8.75	0.50	$(Co)(Pyr)_2$	0.44	2.00
			$(Fe)(Pyr)$	4.83	1.00	$(Co)(Lac)$	1.92	0.10
			$(Fe)_2(Pyr)_2$	5.52	1.00	$(Co)(Lac)_2$	2.97	0.10
			$(Fe)(Gly)$	10.83	0.10	$(Co)(Gly)$	5.45	0.10
			$(Fe)(Gly)_2$	20.48	0.10	$(Co)(Gly)_2$	9.48	0.10
			$(Fe)(Cl)$	0.67	0.10	$(Co)(Cl)$	0.6	0.00
			$(Fe)(Cl)_2$	1.37	0.10	$(Co)(Cl)_2$	0.02	0.00

Table S7. Solubility product log*Ksp* of the considered species at 25 °C and various ionic strength *I*. Charges of the species were omitted for simplicity.

**Solid species**		
**Species**	**log*Ksp***	***I*** [mol·L^{-1}]
**(Ca)(OH)$_2$**	22.75	0
**(Mg)(OH)$_2$**	17.11	0
**(Al)(OH)$_3$**	7.75	0
**(Fe)(OH)$_3$**	3.5	0
**(Si(OH)$_4$)**	-3.74	0
**(Co)(OH)$_2$**	13.24	0
**(Ca)(PO$_4$)(H)**	6.6	0
**(Ca)(SO$_4$)**	-4.59	0
**(Ca)(CO$_3$)**	-8.485	0

Table S8. Concentration of components in the leachates of ES1, Qz, and Resin in ALF and GS.

	**ALF**			**GS**		
	**ES1**	**Qz**	**Resin**	**ES1**	**Qz**	**Resin**
	[M]	[M]	[M]	[M]	[M]	[M]
**Na^+**	5.96E-02	5.96E-02	5.96E-02	1.42E-01	1.42E-01	1.42E-01
**K^+**	0	0	0	4.00E-03	4.00E-03	4.00E-03
**Ca^{2+}**	8.71E-04	8.71E-04	8.71E-04	2.50E-03	2.50E-03	2.50E-03
**Mg^{2+}**	5.25E-04	5.25E-04	5.25E-04	9.98E-04	9.98E-04	9.98E-04
**Al^{3+}**	4.08E-05	5.56E-05	3.34E-06	3.34E-06	3.34E-06	3.34E-06
**Si^{4+}**	1.42E-04	1.50E-04	8.90E-06	1.64E-04	1.60E-04	1.42E-05
**Fe^{3+}**	8.24E-05	1.79E-06	9.67E-05	8.95E-07	8.95E-07	8.95E-07
**Co^{2+}**	3.22E-05	5.09E-07	5.09E-07	6.79E-07	5.09E-07	5.09E-07
**Cl^-**	5.77E-02	5.77E-02	5.77E-02	1.14E-01	1.14E-01	1.14E-01
**PO_4^{3-}**	5.00E-04	5.00E-04	5.00E-04	8.88E-04	8.88E-04	8.88E-04
**SO_4^{2-}**	2.75E-04	2.75E-04	2.75E-04	4.44E-04	4.44E-04	4.44E-04
**CO_3^{2-}**	0	0	0	3.10E-02	3.10E-02	3.10E-02
**acetate**	0	0	0	4.04E-03	4.04E-03	4.04E-03
**citrate**	1.09E-01	1.09E-01	1.09E-01	3.30E-04	3.30E-04	3.30E-04
**tartrate**	3.91E-04	3.91E-04	3.91E-04	0	0	0
**pyruvate**	7.82E-04	7.82E-04	7.82E-04	0	0	0
**lactate**	7.59E-04	7.59E-04	7.59E-04	0	0	0
**glycine**	7.86E-04	7.86E-04	7.86E-04	0	0	0

3. Supplemental Figures

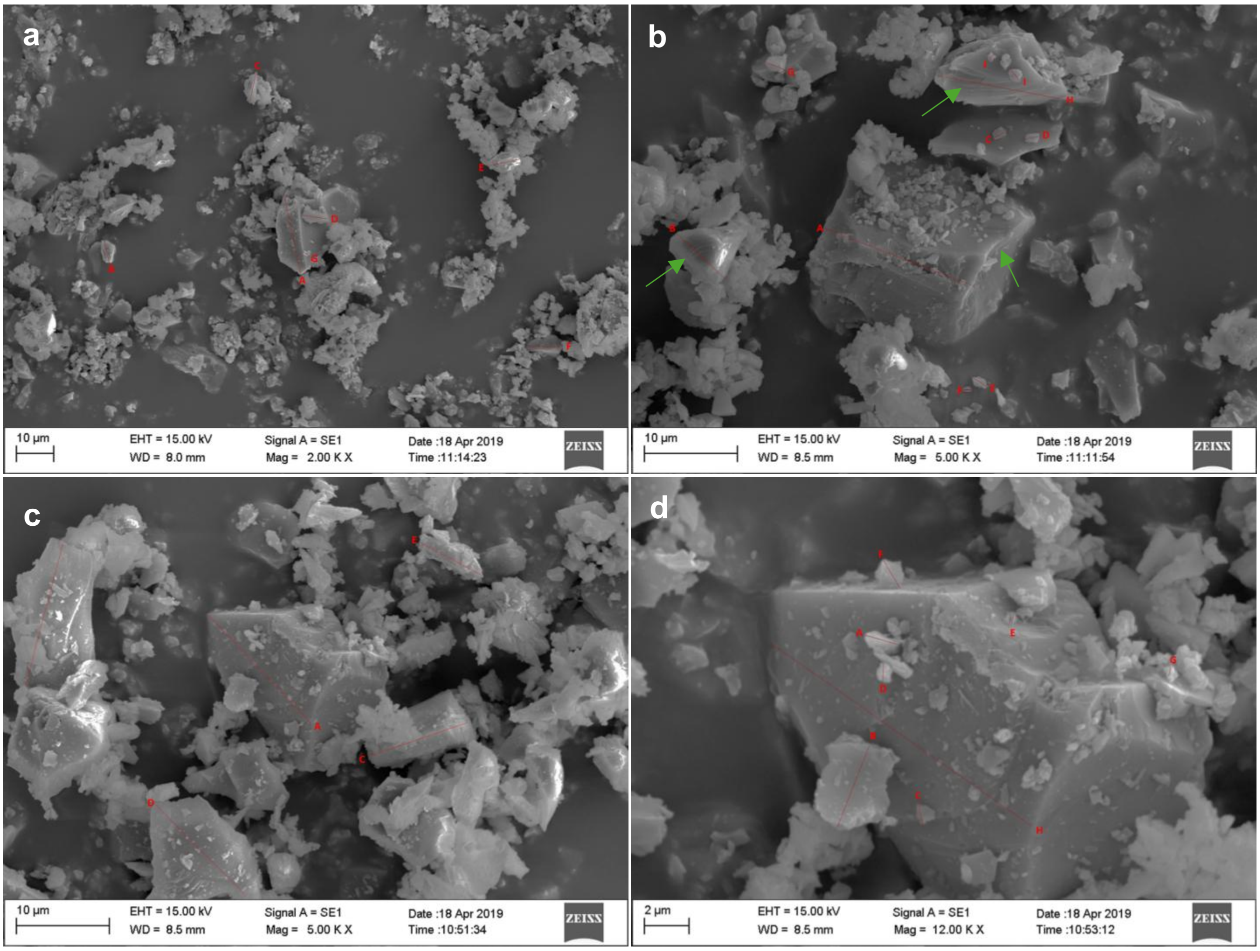

Fig. S1. Micromorphology of ES1 (a, b) and Qz (c, d) obtained by SEM microscopy according to Supplementary methods; magnification: 2,000× (a), 5,000× (b, c), 12,000× (d). Conchoidal fractures are evidenced by green arrows.

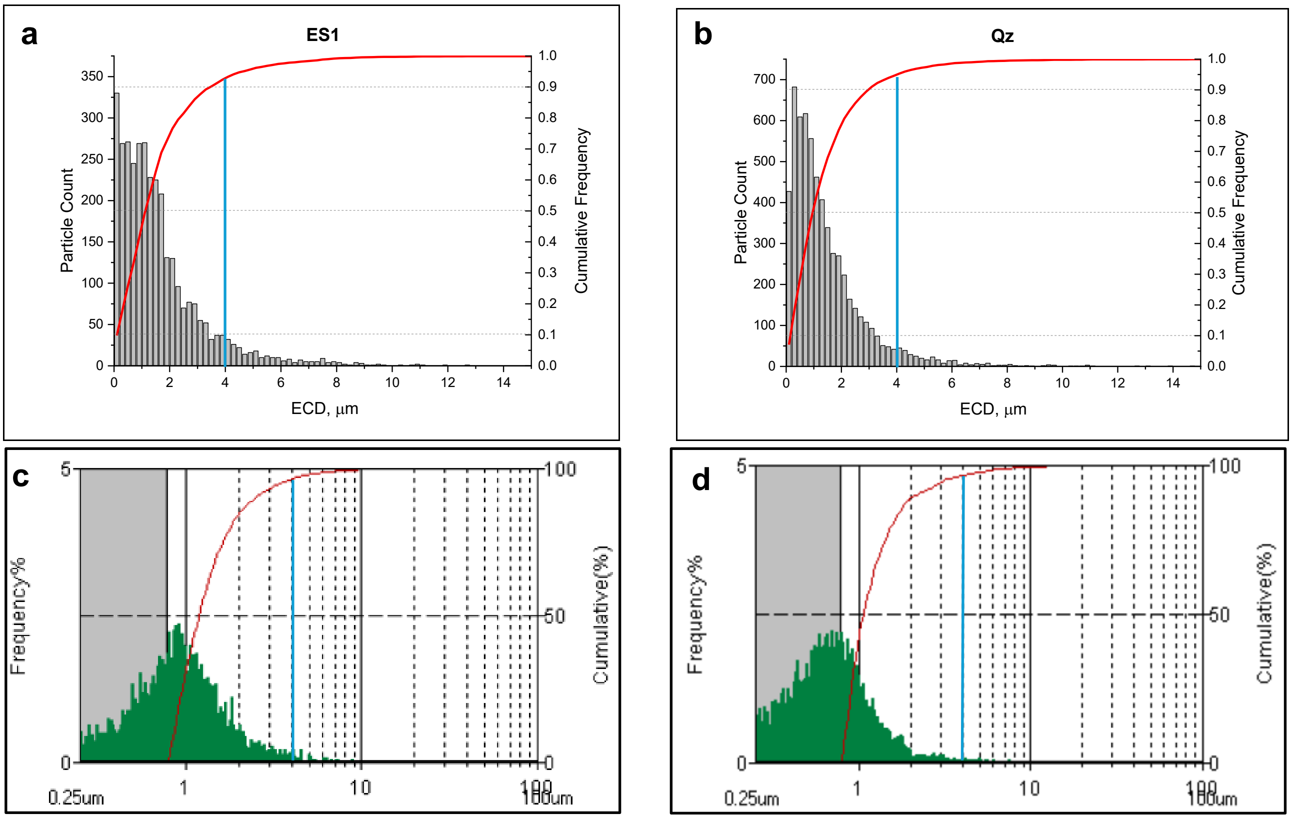

e	Mean ECD (µm ± SD)
ES1	1.55 ± 1.07
Qz	1.45 ± 1.37
rQz	1.93 ± 0.94

Fig. S2. Particle size of ES1, Qz and reference quartz (rQz) determined by automated SEM analysis coupled with AZtecFeature suite (a, b) or particles suspended in water as determined by FPIA (c, d, e) as indicated in the Supplementary methods. (a, b) Most of the particles (~95%) exhibited an equivalent circular diameter (ECD) of less than 4 µm, which falls within the respirable aerodynamic diameter range.[9] All particles had an ECD below 10 µm, corresponding to the thoracic fraction in terms of aerodynamic diameter.[9]

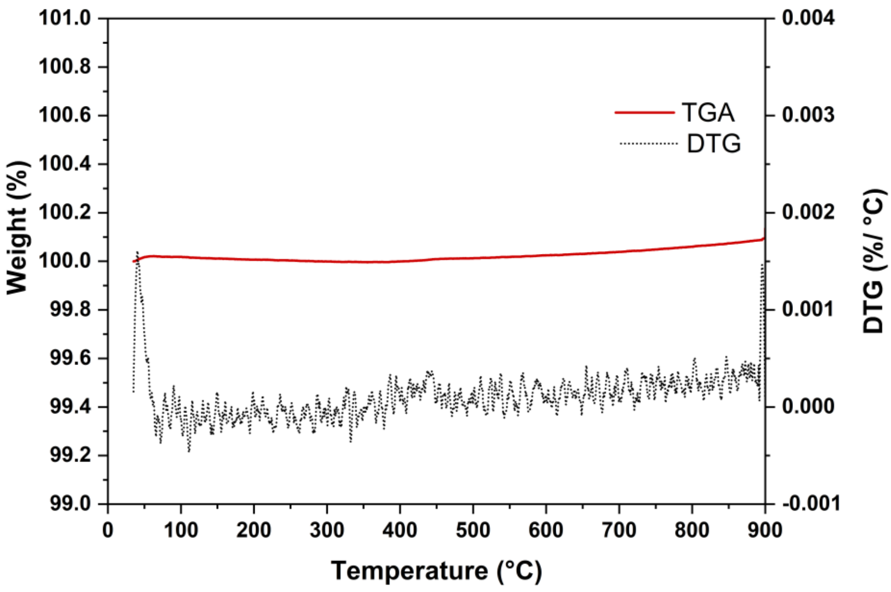

Fig. S3. Thermogram (solid red line) and derivative curve (dotted black line) of pure quartz (Qz) obtained by TGA analysis in artificial air.

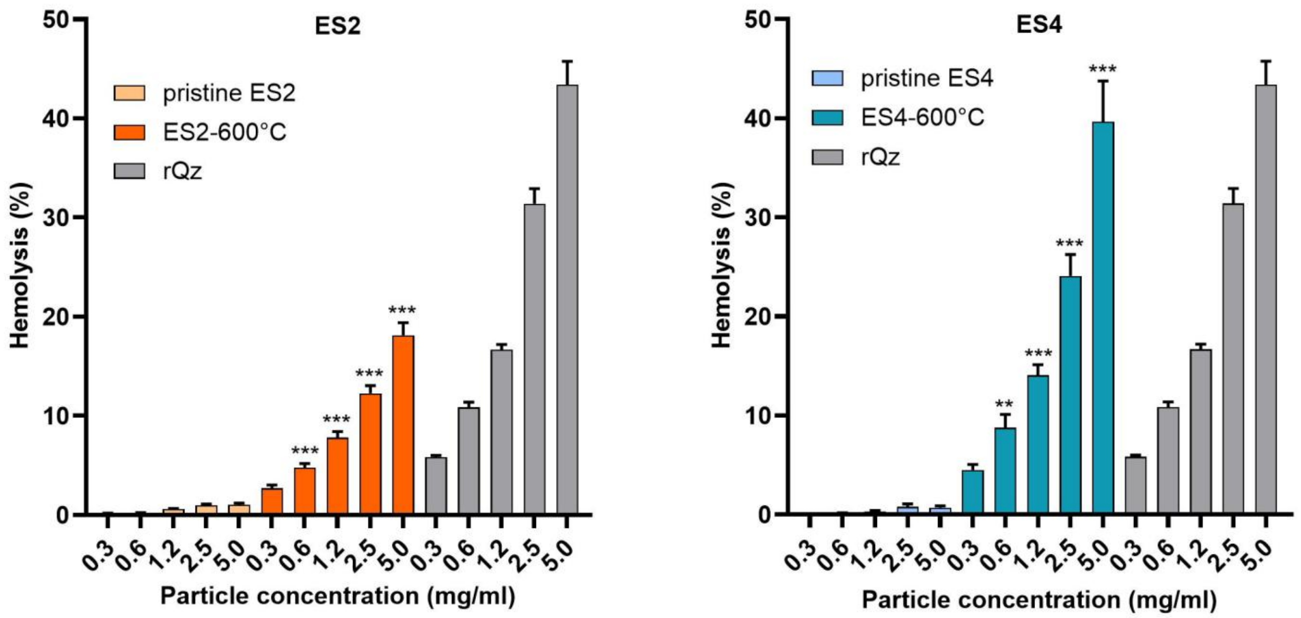

Fig. S4. Membranolytic activity (hemolysis, %) at increasing particle concentration (mg/ml) of pristine ES2 or ES4 dust, both heated at 600 °C for 2h, and a reference quartz (rQz). Data are mean ± SEM of three independent experiments. One-way ANOVA with Šídák's *post hoc* test was applied to compare pristine *vs* heated samples for each dose, $^{**}p < 0.01$ and $^{***}p < 0.001$

a

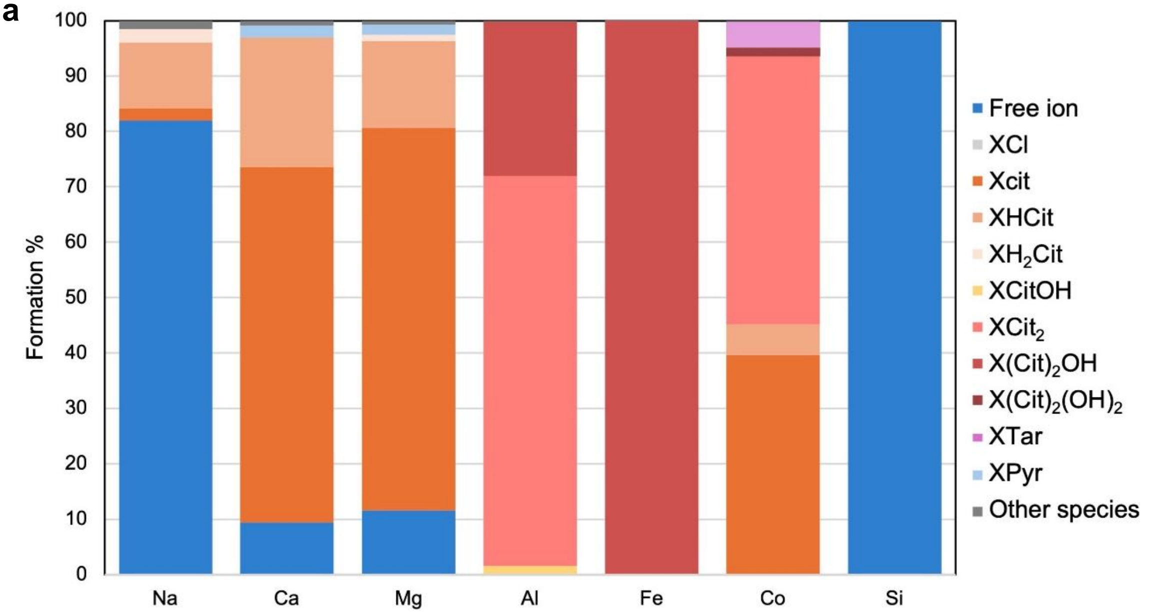

b

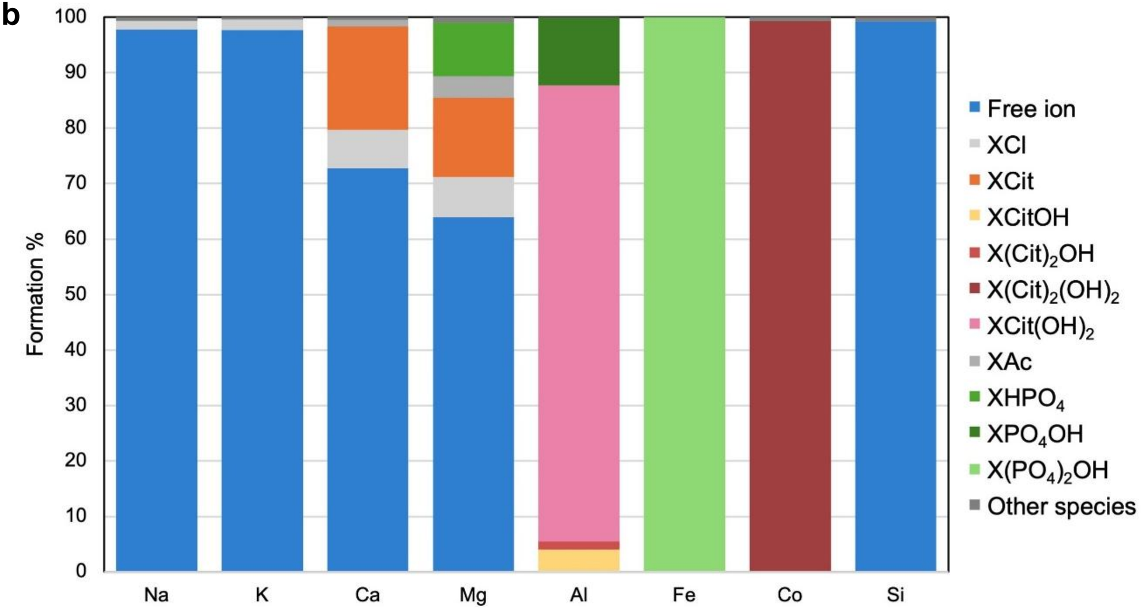

Fig. S5. Element distribution among the different chemical species, expressed as percentage, for ES1 leachate in ALF at pH 4.5 (a) and in GS at pH 7.4 (b). Charges of the species were omitted for simplicity. In the leachate from ES1 in ALF (pH 4.5), the cations (Ca^{2+}, Mg^{2+}, Al^{3+}, Fe^{3+}, and Co^{2+}) were predominantly present as citrate complexes, often accounting for more than 90% of each element speciation. Sodium existed mainly as free ion, while silicon was almost entirely present as $Si(OH)_4$(aq). The speciation pattern in GS (pH 7.4) was markedly different from ALF. Alkali and alkaline earth metals were mostly found as free cations. Al^{3+} and Co^{2+} were chelated by citrate, forming $[AlCit(OH)_2]^{2-}$ and $[Co(Cit)_2(OH)_2]^{6-}$, respectively. Silicon occurred as $Si(OH)_4$(aq), as in ALF. In contrast to ALF, Fe^{3+} predominantly interacted with PO_4^{3-}, likely due to the high abundance of this anion in GS.

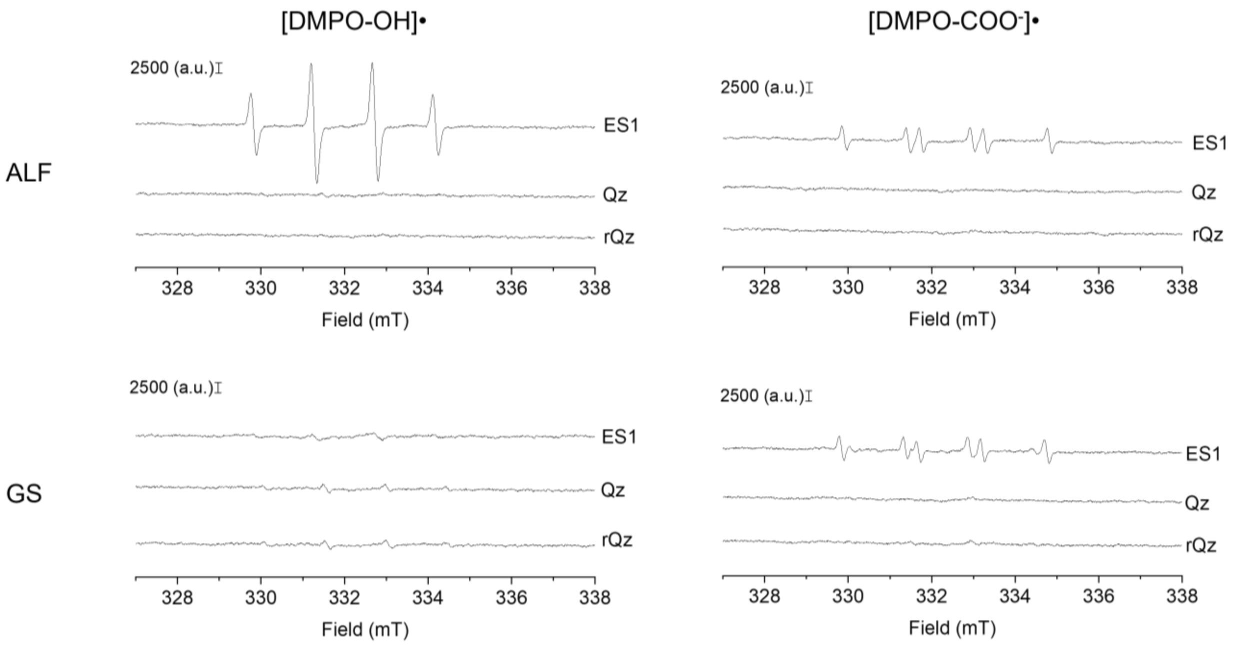

Fig. S6. EPR spectra of the [DMPO-OH]$^{\cdot}$ or [DMPO-COO]$^{\cdot-}$ adducts recorded after 10 min of incubation of ES1, pure quartz (Qz), or a reference quartz (rQz) with ALF or GS, DMPO and H2O2 or sodium formate. The spectra reported represent a single measurement, selected as representative of multiple measurements.

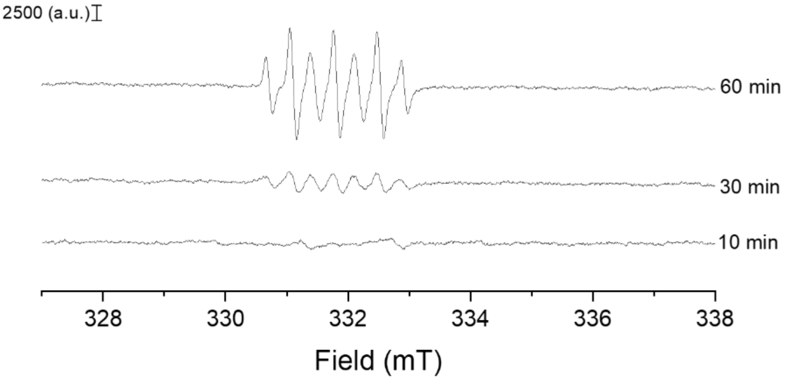

Fig. S7. EPR spectra recorded after 10, 30 and 60 min of ES1 incubation in GS, DMPO and H2O2. The spectra reported represent a single measurement, selected as representative of multiple measurements**.** According to the line shape and the hyperfine splittings, the spectra at 30 and 60 min are assigned to 5,5-dimethylpyrrolidone-(2)-oxy-(1) (DMPOX). [10]

4. Supplemental references

1. Castellino, L.; Alladio, E.; Bertinetti, S.; Lando, G.; De Stefano, C.; Blasco, S.; García-España, E.; Gama, S.; Berto, S.; Milea, D., PyES – An open-source software for the computation of solution and precipitation equilibria. *Chemometr. Intell. Lab. Syst.* **2023,** *239*, 104860.

2. Brown, P.L.; Ekberg, C., *Hydrolysis of Metal Ions*, Wiley, **2016**; 620–628. DOI: 10.1002/9783527656189.

3. Thoenen, T.; Hummel, W.; Berner, U.; Curti, E., The PSI/Nagra Chemical Thermodynamic Database 12/07. Paul Scherrer Institut, Villigen PSI, Switzerland, **2014**, pp. 205−212.

4. Hummel, W.; Thoenen, T., Technical Report 21-03. The PSI Chemical Thermodynamic Database 2020. NAGRA, Ed. Wettingen, **2023**.

5. Baes, C. F.; Mesmer, R. S., The Hydrolysis of Cations. *Berichte der Bunsengesellschaft für physikalische Chemie* **1977,** *81* (2), 245-246, pp. 252-259.

6. The IUPAC Stability Constants Database, Sc-Database, https://www.acadsoft.co.uk/, accessed: 2025/07/10

7. Foti, C.; Gianguzza, A.; Sammartano, S., A comparison of equations for fitting protonation constants of carboxylic acids in aqueous tetramethylammonium chloride at various ionic strengths. *J. Solut. Chem.* **1997,** *26* (6), 631-648.

8. Kumarasamy, C.; Pisaniello, D.; Gaskin, S.; Hall, T., What do safety data sheets for artificial stone products tell us about composition? A comparative analysis with physicochemical data. *Ann. Work Expo. Health* **2022,** *66* (7), 937-945.

9. Brown, J. S.; Gordon, T.; Price, O.; Asgharian, B., Thoracic and respirable particle definitions for human health risk assessment. *Part. Fibre Toxicol.* **2013,** *10* (1), 12.

10. Leonard, S.; M. Gannett, P.; Rojanasakul, Y.; Schwegler-Berry, D.; Castranova, V.; Vallyathan, V.; Shi, X., Cobalt-mediated generation of reactive oxygen species and its possible mechanism. *J. Inorg. Biochem.* **1998,** *70* (3), 239-244.
